# BRAF and AXL oncogenes drive RIPK3 expression loss in cancer

**DOI:** 10.1371/journal.pbio.2005756

**Published:** 2018-08-29

**Authors:** Ayaz Najafov, Ioannis K. Zervantonakis, Adnan K. Mookhtiar, Patricia Greninger, Ryan J. March, Regina K. Egan, Hoang Son Luu, Daniel G. Stover, Ursula A. Matulonis, Cyril H. Benes, Junying Yuan

**Affiliations:** 1 Department of Cell Biology, Harvard Medical School, Boston, Massachusetts, United States of America; 2 Ludwig Center, Harvard Medical School, Boston, Massachusetts, United States of America; 3 Massachusetts General Hospital Cancer Center, Boston, Massachusetts, United States of America; 4 Division of Medical Oncology, Ohio State University Comprehensive Cancer Center, Columbus, Ohio, United States of America; 5 Gynecologic Oncology Program, Department of Medical Oncology, Dana-Farber Cancer Institute, Boston, Massachusetts, United States of America; 6 Department of Medicine, Harvard Medical School, Boston, Massachusetts, United States of America; St. Jude Childrens Research Hospital, Memphis, United States of America

## Abstract

Necroptosis is a lytic programmed cell death mediated by the RIPK1-RIPK3-MLKL pathway. The loss of Receptor-interacting serine/threonine-protein kinase 3 (RIPK3) expression and necroptotic potential have been previously reported in several cancer cell lines; however, the extent of this loss across cancer types, as well as its mutational drivers, were unknown. Here, we show that RIPK3 expression loss occurs progressively during tumor growth both in patient tumor biopsies and tumor xenograft models. Using a cell-based necroptosis sensitivity screen of 941 cancer cell lines, we find that escape from necroptosis is prevalent across cancer types, with an incidence rate of 83%. Genome-wide bioinformatics analysis of this differential necroptosis sensitivity data in the context of differential gene expression and mutation data across the cell lines identified various factors that correlate with resistance to necroptosis and loss of RIPK3 expression, including oncogenes BRAF and AXL. Inhibition of these oncogenes can rescue the RIPK3 expression loss and regain of necroptosis sensitivity. This genome-wide analysis also identifies that the loss of RIPK3 expression is the primary factor correlating with escape from necroptosis. Thus, we conclude that necroptosis resistance of cancer cells is common and is oncogene driven, suggesting that escape from necroptosis could be a potential hallmark of cancer, similar to escape from apoptosis.

## Author summary

Necroptosis is a regulated process that triggers cell death, resulting in necrosis and inflammation. Cancer cells have been shown to lose their ability to die via necroptosis, but the genetic factors that drive this resistance remain unknown. Here, we have analyzed 941 different cancer cell types and found that 83% of them are fully resistant to necroptosis. In order to identify the mechanisms underlying necroptosis resistance in these cells, we performed bioinformatics analyses to identify genes whose overexpression or mutation correlate with this effect. We show that two major genes, which are frequently deregulated in cancer (also known as oncogenes), are key drivers of the resistance to necroptosis, and that targeting these oncogenes with specific drugs reversed this resistance. We conclude that resistance to necroptosis is a common event in cancer that can be overcome by targeting the genes that drive this resistance, which subsequently allows stimulation of cancer cell death via necroptosis.

## Introduction

Necroptosis is a necrotic programmed cell death pathway mediated by the RIPK1-RIPK3-MLKL signaling cascade [1–4]. Receptor-interacting serine/threonine-protein kinase 1 (RIPK1) can be activated when cells are stimulated by Tumor necrosis factor alpha (TNFα), Fas, or TRAIL ligands as well as downstream of Toll-like receptors [5,6]. Cells can be sensitized to necroptosis by repressing function of the inhibitor of apoptosis proteins (IAPs: cIAP1, cIAP2, and XIAP) by Smac mimetics, such as SM-164, while caspase inhibition by a pan-caspase inhibitor such as zVAD.fmk also further sensitizes cells to necroptosis [5,7,8].

During necroptosis activation, RIPK1 interacts with Receptor-interacting serine/threonine-protein kinase 3 (RIPK3) to form the necrosome, which in turn phosphorylates pseudokinase Mixed lineage kinase domain-like protein (MLKL) to mediate necrotic cell death via plasma membrane rupture [9–17]. In addition to necroptosis [9,17–21], RIPK3 has been implicated in regulation of antitumor immunity [22], apoptosis [6,11,23–29], and cytokine production [30,31]. While RIPK3 expression has been shown to be lost in several cancer cell lines and cancer types [18,21,32–34], no systematic evidence for the extent of this loss across cancer types or the mechanisms driving this loss have been reported.

The Tyro3, Axl, Mer (TAM) receptor family of tyrosine kinases plays a role in regulating cell growth, survival, and proliferation [35,36]. TAM kinases are oncogenes, frequently amplified in a variety of cancers, in which their overexpression correlates with poor patient survival [36–40]. Importantly, while TAM kinases are anti-apoptotic and are established as important mediators of resolution of inflammation [41], their roles in the context of necroptosis have not been studied.

BRAF is a major regulator of protein synthesis, cell survival, growth, and proliferation [42]. Overactivation of BRAF is observed in a vast majority of cancers [42–45]. Importantly, while BRAF is an established anti-apoptotic kinase, its role in the regulation of necroptosis is unknown.

In this study, we performed a necroptosis sensitivity screen in 941 human cancer cell lines to identify the mutational drivers of the RIPK3 expression loss and the consequent escape from necroptosis. We identified the oncogenic kinases BRAF and AXL, which were validated as potential mediators of this process, because their inhibition can rescue the loss of RIPK3 expression and result in regain of sensitivity to necroptosis. Interestingly, our tumor xenograft studies, as well as transcriptomics analyses of published RNAseq/microarray datasets of patient tumor biopsy samples, show that RIPK3 expression is lost progressively during tumorigenesis. Our results reveal a potential role of BRAF and AXL oncogenes in driving the loss of RIPK3 expression and escape from necroptosis in various cancers.

## Results

### Necroptosis in tumors and the loss of RIPK3 expression during tumor progression

**Fig 1:**
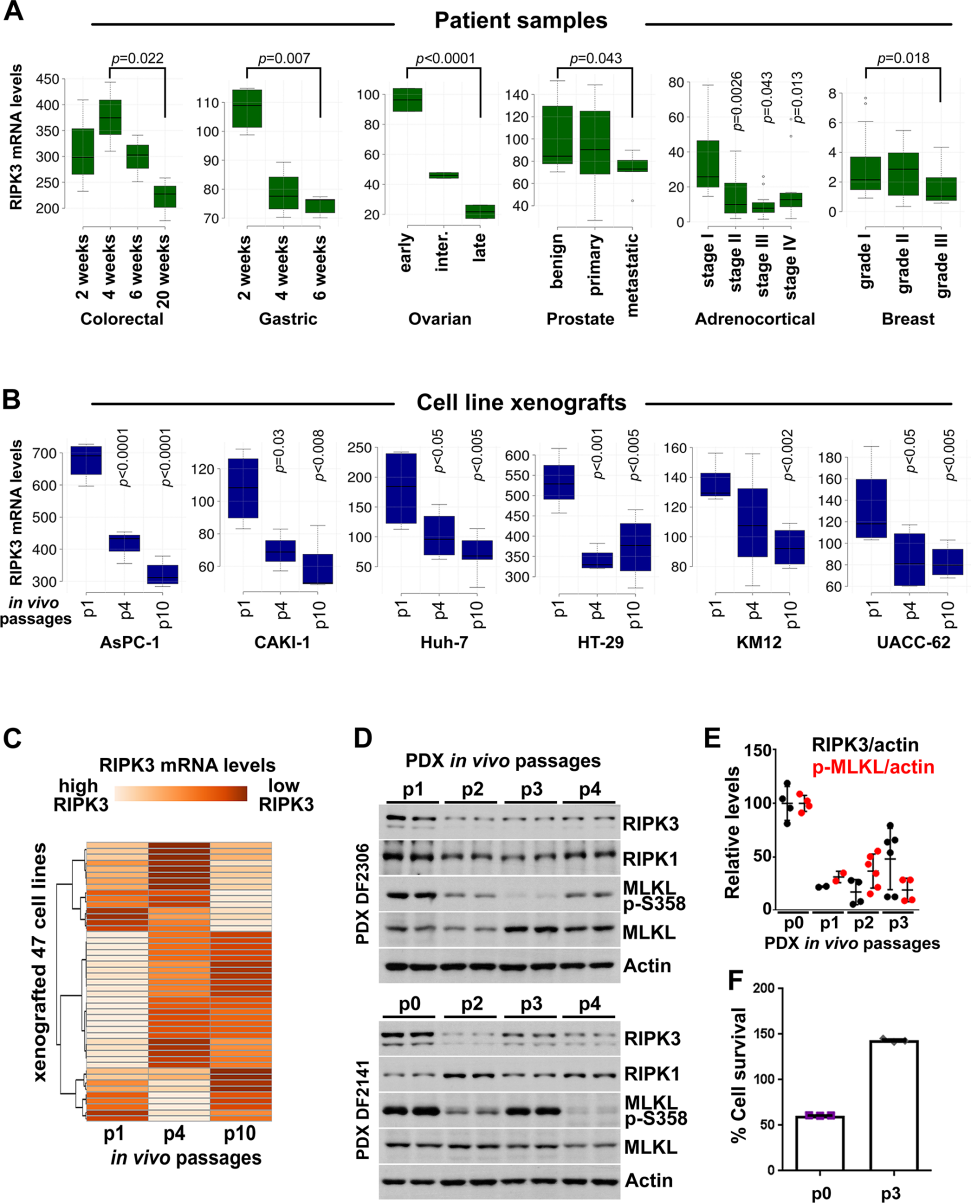
Necroptosis is induced in tumors in vivo and RIPK3 expression is progressively lost during tumorigenesis. **(A)** RIPK3 mRNA levels are decreased during progressive stages of colorectal, gastric, ovarian, prostate, adrenocortical, and breast cancers. The results shown here are in part based upon data generated by the TCGA Research Network. See S6 Table for details about the studies. **(B)** RIPK3 expression is lost during progressive in vivo passages of mouse tumor xenografts of indicated cancer cell lines. **(C)** As in (B), except data from 47 different cell lines are presented as a heatmap. **(D)** Necroptosis is induced in tumors in vivo and RIPK3 expression is progressively lost during tumorigenesis. Necroptosis induction is determined by the MLKL p-S358. Ovarian PDX lysates obtained at the indicated in vivo passages were immunoblotted with the indicated antibodies. **(E)** Quantification of the RIPK3 and p-MLKL levels shown in (D) and their normalization to actin. **(F)** Ovarian PDX cells at indicated in vivo passages were cultured and treated with TSZ to induce necroptosis. Cell survival was determined 24 hours after treatment, using CellTiterGlo. The underlying data can be found in S1 Data. PDX, patient-derived xenograft; p-MLKL, phospho-MLKL S358; TSZ, TNFα+SM-164+zVAD.fmk.

In order to understand the relevance of necroptosis in tumor growth and the in vivo kinetics of the RIPK3 expression loss during tumorigenesis, we evaluated the changes in RIPK3 mRNA levels in published transcriptomics datasets. Six patient tumor biopsy studies [46–51] and one cancer cell line xenograft study [52] were analyzed. We found that RIPK3 mRNA levels were progressively lost during tumor growth in colorectal, gastric, and ovarian cancer patients (Fig 1A). Notably, the loss of RIPK3 expression also associated with the progression to metastasis in human prostate tumors, and higher-grade adrenocortical and breast tumors (Fig 1A). Moreover, RIPK3 expression was also progressively lost during in vivo passaging of tumor xenografts using 47 human cancer cell lines, in which the majority of the cell lines showed a strong loss of RIPK3 expression at passage 10, compared to passage 1, with some heterogeneity in the extent of the loss in a fraction of the cell lines (Fig 1B and 1C).

Because the most robust RIPK3 expression loss was observed in ovarian cancer biopsies (Fig 1A), we performed a newly derived patient-derived xenograft (PDX) study using primary cells obtained from high-grade serous ovarian cancer biopsies, in order to determine whether necroptosis is physiologically activated in tumors and whether RIPK3 protein levels indeed are lost during tumorigenesis progression. We found that the expression of RIPK3, but not that of RIPK1, was progressively reduced during xenograft tumor growth in four out of five PDX samples derived from high-grade serous ovarian cancer biopsies (Fig 1D and 1E, S1A Fig). In addition, we found that MLKL was phosphorylated at Ser358 in tumors at early in vivo xenograft passages (passage 0), revealing that the necroptosis pathway is endogenously activated in tumors. Consistent with the loss of RIPK3 expression, MLKL phospho-Ser358 levels decreased as a function of serial in vivo passage of the PDXs (Fig 1D and 1E). Importantly, while ex vivo–cultured tumor xenograft cells were sensitive to TNFα+SM-164+zVAD.fmk (TSZ)-induced necroptosis at passage zero, they were fully resistant after the third in vivo serial xenograft, and because of the resistance to cell death, this treatment of TSZ did not induce cell death, but rather induced cell growth resulting in an approximately 140% survival rate (Fig 1F and S1B Fig). The TSZ-induced necroptosis in these cells was potently blocked by 10 μM of the RIPK1 inhibitor Nec-1s and 10 μM of the RIPK3 inhibitor GSK’872, and was also blocked by 10 μM of the MLKL inhibitor necrosulfonamide (NSA) (S1B and S1C Fig).

These findings reveal that the loss of RIPK3 expression occurs progressively during tumorigenesis in vivo and that necroptosis is activated in tumors that express RIPK3.

### High-throughput necroptosis sensitivity screen reveals a prevalent loss of necroptosis potential in cancer cell lines

**Fig 2:**
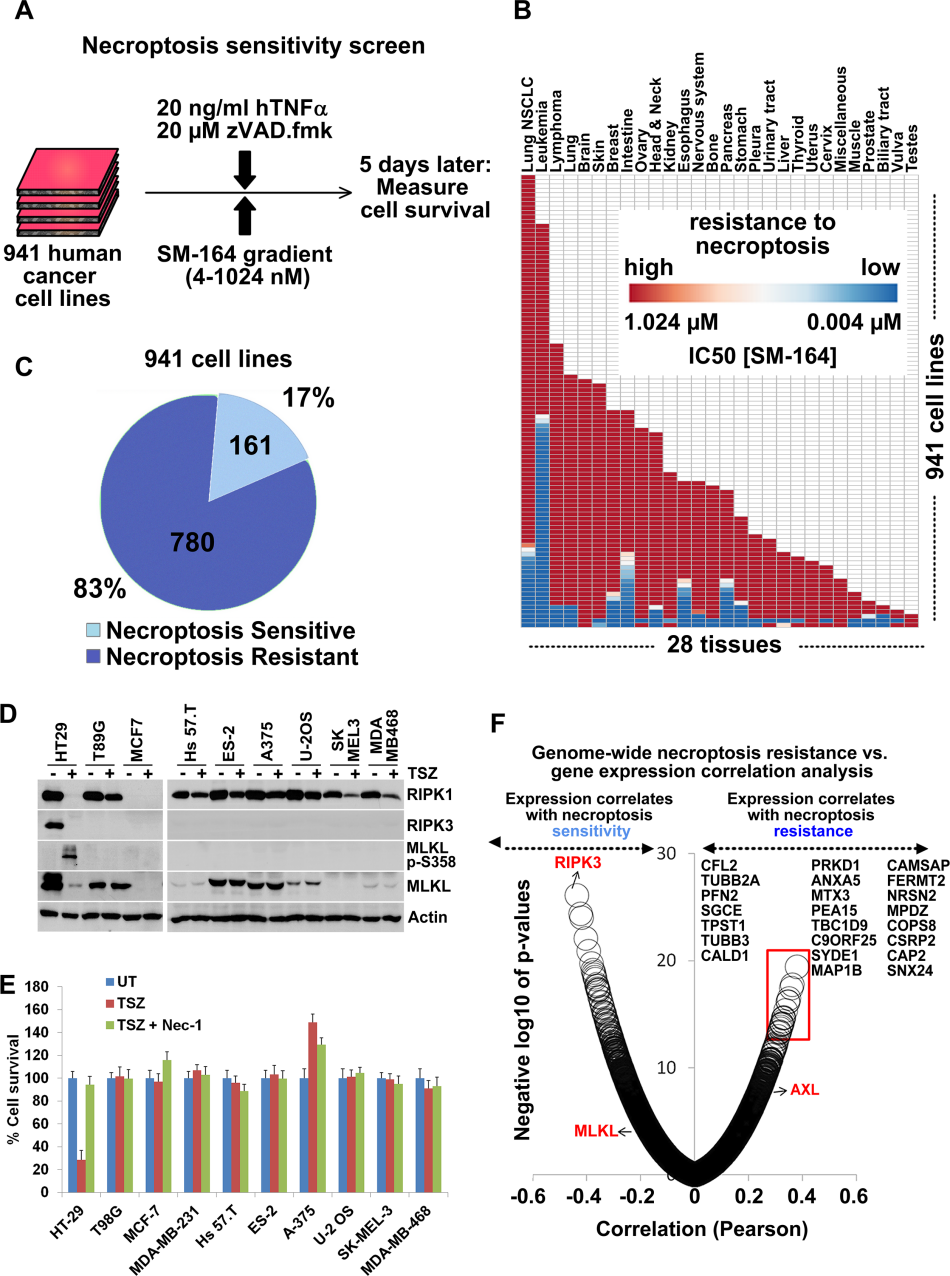
Necroptosis sensitivity screen in 941 cancer cell lines identifies drivers of necroptosis resistance. **(A)** Outline of the high-throughput screening for differential necroptosis sensitivity in 941 human cancer cell lines. **(B)** Differential sensitivity of 941 cancer cell lines to TSZ-induced necroptosis across 28 tissues of origin. **(C)** Numbers and percentages of necroptosis-resistant/sensitive cell lines. **(D)** Low-throughput confirmation of the screen observations regarding loss of RIPK3 expression and necroptosis resistance, as judged by lack of p-MLKL induction. Indicated cancer cell lines were treated with TSZ for 6 hours and cell lysates were immunoblotted with indicated antibodies. Note that RIPK1, RIPK3, and MLKL levels decrease in lane 2 because of induction of necroptosis, formation of amyloid-like necrosome structure, and translocation of these proteins into a detergent-insoluble fraction. **(E)** Low-throughput confirmation of the screen observations regarding necroptosis resistance. Indicated cells were treated with indicated treatments and cell survival was measured 16 hours later using CellTiterGlo. Means ± SEM are shown. **(F)** Genome-wide Pearson correlation analysis of TSZ-IC_50_ values versus gene expression values across 941 cell lines identifies genes, the expression of which negatively (e.g., RIPK3) and positively (e.g., AXL) correlates with necroptosis resistance. Top genes, the expression of which positively correlates with necroptosis resistance (red box), are listed. The underlying data can be found in S1 Data. p-MLKL, phospho-MLKL S358; TSZ TNFα+SM-164+zVAD.fmk; UT, untreated.

In order to identify the mechanisms driving RIPK3 expression loss in cancer cells, we performed a necroptosis sensitivity screen using a panel of 941 human cancer cell lines from the Genomics of Drug Sensitivity in Cancer (GDSC) collection, which represent various cancer types from 28 tissues [53,54]. A potent TNFα + SM-164 + zVAD.fmk (TSZ) treatment was used to stimulate necroptotic cell death under nine different SM-164 concentration conditions in the 4–1,024 nM range (Fig 2A). Remarkably, we found that 780 (83%) of these cell lines were fully resistant to necroptosis induced by TSZ even at the highest SM-164 concentration (Fig 2B and 2C, S1 and S2 Tables). These screen results were validated by testing 23 randomly selected cancer cell lines, which showed a complete resistance to TSZ- and TNFα+Cycloheximide+zVAD.fmk (TCZ)-induced necroptosis, lack of RIPK3 expression, and lack of MLKL Ser358 phosphorylation upon stimulation with TSZ treatment (Fig 2D and 2E and S2A Table). Out of 28 tissue types from which the cancer cell lines were derived, 8 tissue types were found to have no sensitive cell lines, and no tissue type was found to lack resistant lines (S2B and S2C Fig).

Together, these results suggest that the escape from necroptosis is found in most cancer cell lines, independent of tissue and cancer type.

### AXL overexpression in cancer promotes the loss of RIPK3 expression

Having established that RIPK3 expression loss is observed during tumorigenesis (Fig 1) and that this loss is prevalent across cancer types (Fig 2B), we next set out to identify drivers of this loss. We performed genome-wide Pearson correlation analysis using the mRNA expression datasets from both GDSC and Broad-Novartis Cancer Cell Line Encyclopedia [55] (CCLE) in order to identify genes whose elevated expression correlates with high TSZ-IC_50_ values (i.e., resistance to necroptosis). We used both databases because the GDSC and the CCLE database cell line collections overlap and the expression values obtained from two independent sources would increase the confidence in the obtained correlation results. Our correlation analyses revealed 634 genes whose expression positively correlated with the resistance to necroptosis (*p* < 0.01, Bonferroni correction). RIPK3 expression was the most negatively correlated with resistance to necroptosis (Pearson coefficient = −0.43, *p* = 4.11 × 10^−24^) and its low expression was significantly enriched in necroptosis-resistant (NR) cell lines, confirming the validity of the screen and the analysis strategy (Fig 2F and S3A Fig). Consistently with its key role in necroptosis, MLKL expression also negatively correlated with resistance to necroptosis (Pearson coefficient = −0.25, *p* = 8.45 × 10^−7^), while RIPK1 expression did not (Fig 2F). Importantly, 20 of these genes were known to be classified as oncogenes or genes that promote oncogenic transformation (see Materials and methods for the bioinformatics analysis description) (S3B Fig).

**Fig 3:**
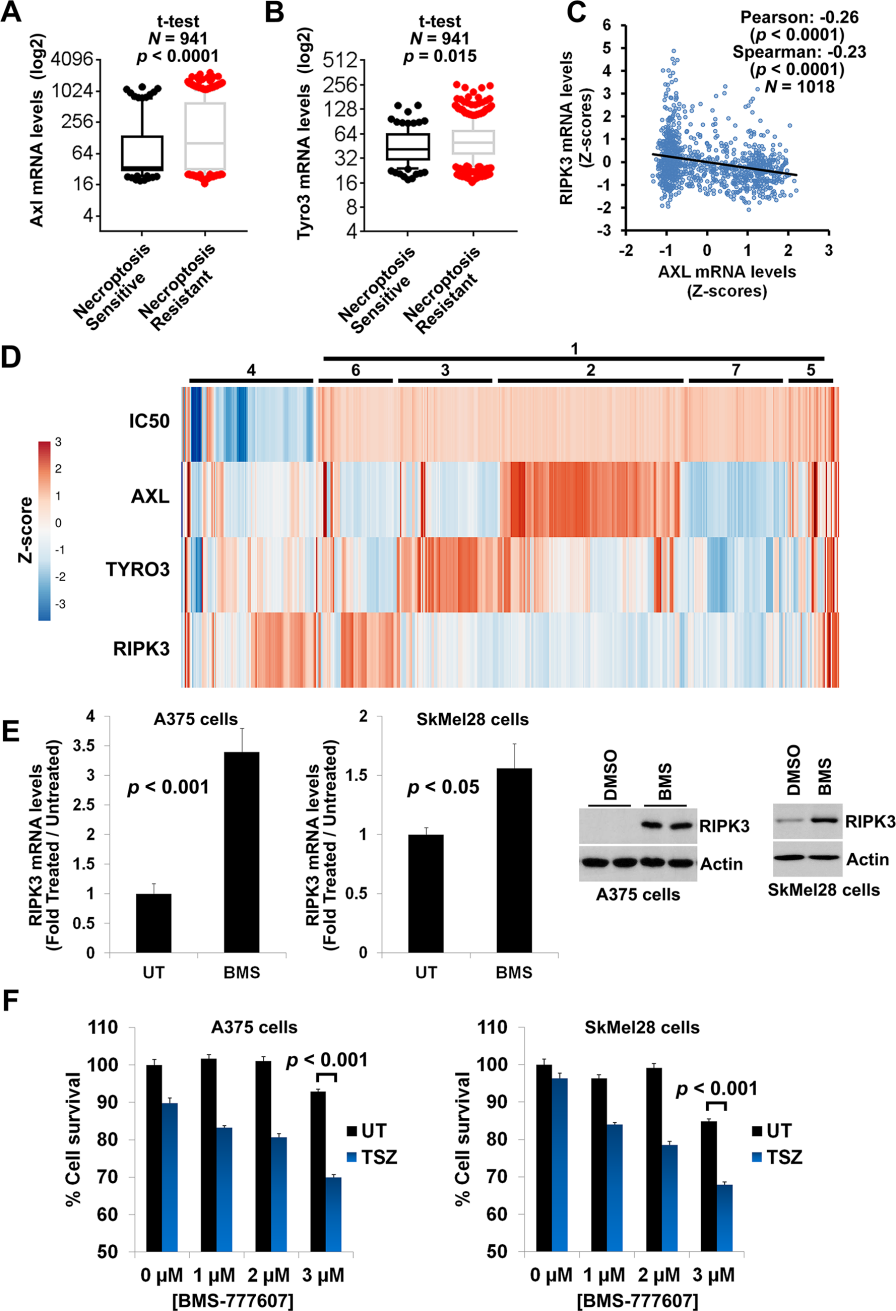
AXL overexpression in cancer cell lines correlates with loss of RIPK3 expression and gain of necroptosis resistance. **(A)** High AXL expression levels are enriched in cancer cell lines fully resistant to necroptosis. GDSC database was employed for the analysis. Means, 10–90 percentile data points ± SEM are shown with *t* test *p*-values. **(B)** High TYRO3 expression levels are enriched in cancer cell lines fully resistant to necroptosis. GDSC database was employed for the analysis. Means, 10–90 percentile data points ± SEM are shown with *t* test *p*-values. **(C)** High AXL expression predicts low RIPK3 expression levels. GDSC database was employed for the Pearson and Spearman correlation analyses. **(D)** High AXL/TYRO3 expression positively correlates with low RIPK3 expression and high TSZ-IC_50_ levels (resistant to necroptosis). Heatmap showing clustering of z-score values for TSZ-IC_50_ versus AXL, TYRO3, and RIPK3 expression levels across 941 cell lines. Numbers indicate clusters described in the text. **(E)** Inhibition of AXL in cancer cell lines can rescue loss of RIPK3 expression. qRT-PCR and western blotting analysis of RIPK3 expression in A375 and SkMel28 cell lines following 4 days of AXL inhibition by 1 μM of BMS-777607. These cell lines were selected because they did not show significant cell death following treatment with this inhibitor. **(F)** Inhibition of AXL in cancer cell lines can rescue loss of necroptosis sensitivity. A375 and SkMel28 cells were treated with indicated concentrations of BMS-777607 for 4 days. Drugs were washed out and necroptosis was induced by 24 h treatment with 25 ng/mL TNFα + 0.5 μM SM-164 + 30 μM zVAD.fmk. Cell survival was determined using CellTiterGlo assay. The underlying data can be found in S1 Data. GDSC, Genomics of Drug Sensitivity in Cancer; qRT-PCR, quantitative real-time PCR; TSZ, TNFα+SM-164+zVAD.fmk; UT, untreated.

Out of the 20 oncogene-related genes, we focused our subsequent experiments on AXL, because (a) its family member TYRO3 was also among the 634 genes that positively correlate with resistance to necroptosis; (b) out of the two TAM kinase family members, AXL expression showed the strongest positive correlation with TSZ-IC_50_ (AXL: Pearson coefficient = 0.21, *p* = 2.91 × 10^−5^; TYRO3: Pearson coefficient = 0.10, *p* = 0.017); and (c) AXL is the predominant TAM kinase family member that is frequently overexpressed in cancer. Importantly, transcriptomics analysis of the screened 941 cancer cell lines revealed that high AXL and TYRO3 mRNA levels predict both resistance to necroptosis and low RIPK3 mRNA levels (Figs 2F and 3A–3D, S3 Table), but not those of RIPK1, MLKL, or any other pro-necroptotic genes (S4A Fig).

AXL expression levels also negatively correlated with RIPK3 expression in stomach adenocarcinoma tumors and acute myeloid leukemia (S4B Fig), based on the analysis of the Cancer Genome Atlas (TCGA) database [56] using cBio Cancer Genomics Portal [51] and according to both Pearson and Spearman correlation analyses.

A similar positive correlation between AXL expression and TSZ-IC_50_, as well as a negative correlation between AXL-RIPK3 expression levels, was observed when expression values from the CCLE database were used for the analysis (S5A and S5B Fig). Quartile analysis of the data also confirmed these Pearson correlation observations (S5C and S5D Fig).

Clustering analysis (Fig 3D) revealed that majority of the analyzed cancer cell lines that are resistant to necroptosis (high IC_50_, cluster 1, about 83%) are either AXL^high^ (cluster 2, about 28%) or TYRO3^high^ (cluster 3, about 14%), while the majority of those that are sensitive to necroptosis (low IC_50_) are RIPK3^high^ and have low/medium AXL/TYRO3 levels (cluster 4, about 19%). While the majority of the cells in the cluster 4 were RIPK3^high^, a fraction were RIPK3^low^, suggesting that high RIPK3 mRNA levels are not a prerequisite to undergo necroptosis and that sufficient RIPK3 protein is expressed in these cells to undergo necroptotic cell death. However, RIPK3 expression levels were more heterogeneous than the TSZ-IC_50_ values, and about 18% of the cell lines were fully resistant to necroptosis despite the presence of RIPK3 expression (clusters 5 and 6), suggesting that the escape from necroptosis may not be only due to loss of RIPK3 expression. Moreover, not all cell lines with high AXL/TYRO3 levels had lost RIPK3 expression (cluster 5). Additionally, about 14% of the cell lines with low RIPK3 levels and resistance to necroptosis did not have high AXL/TYRO3 levels, suggesting that other RIPK3 loss-driving forces may exist (cluster 7). Overall, this analysis revealed a great degree of heterogeneity in AXL/TYRO3 and RIPK3 expression levels and resistance to necroptosis in the screened lines, as well as the presence of high AXL/TYRO3 and concomitant low RIPK3 expression levels in about 56% of the NR lines, suggesting that high expression levels of AXL/TYRO3 could be potential predictors/biomarkers for loss of RIPK3 expression and necroptosis resistance in cancer.

A 4-day treatment of A375 and SkMel28 cancer cell lines, which have no initial RIPK3 expression (but also no genetic mutations of RIPK3), with low concentrations of AXL/TYRO3 inhibitor BMS-777607 resulted in a regain of RIPK3 expression at both mRNA and protein levels (Fig 3E). Importantly, this treatment also restored the sensitivity of these cells to TSZ-induced necroptosis (Fig 3F).

Overall, these findings suggest that AXL/TYRO3 overexpression, frequently seen in cancers, promotes the loss of RIPK3 expression and escape from necroptosis, which may be reversed upon inhibition of these kinases. Moreover, high AXL/TYRO3 levels are potential predictors/biomarkers for loss of RIPK3 expression and necroptosis resistance in cancer.

### Oncogenic BRAF mutations promote loss of RIPK3 expression

**Fig 4:**
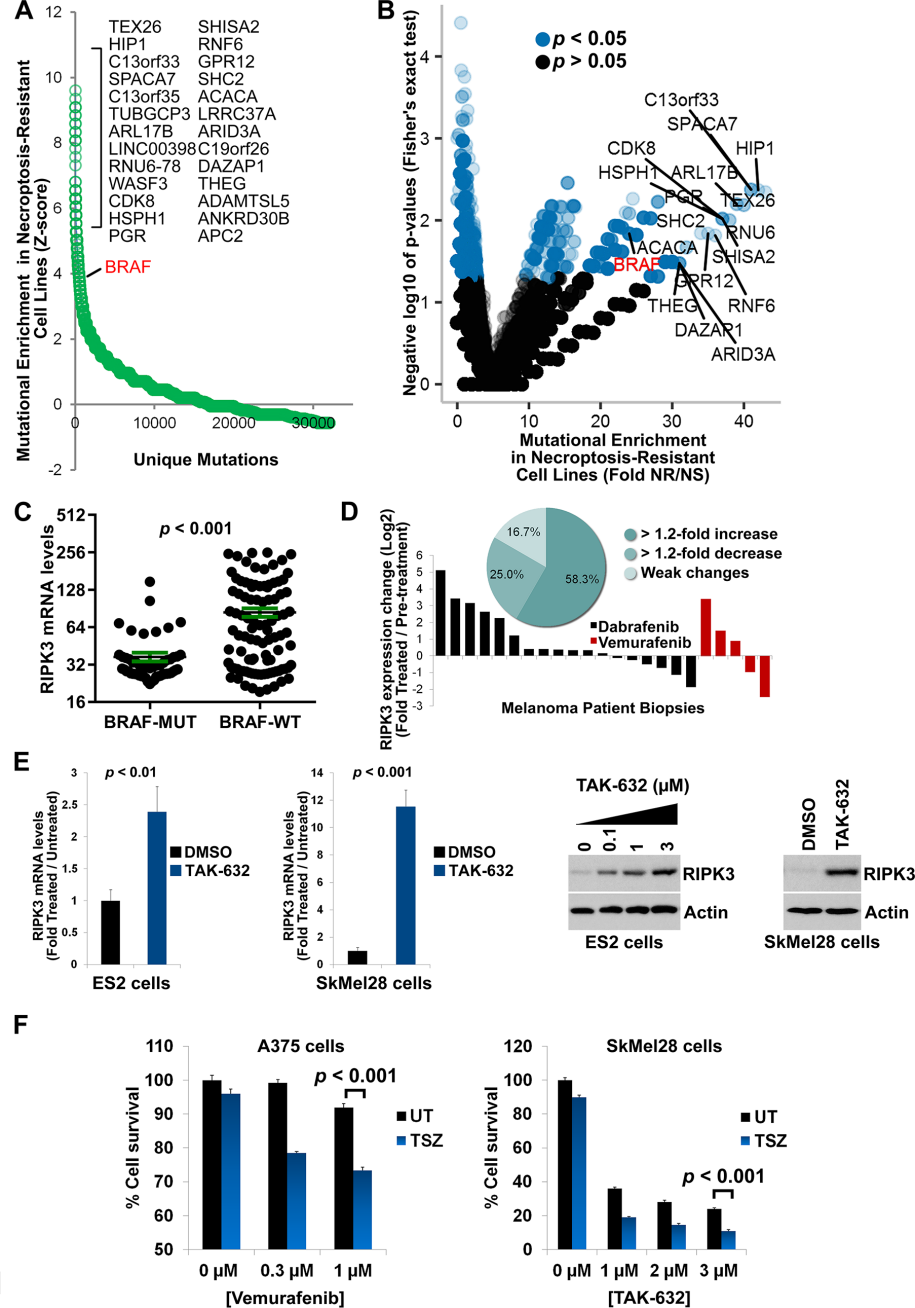
Necroptosis sensitivity screen in 941 cancer cell lines identifies BRAF as a mutational driver of RIPK3 expression loss and gain of necroptosis resistance. **(A)** Mutational drivers of necroptosis resistance in cancer. z-score analysis of the fold mutation enrichment in NR versus NS cancer cell lines. BRAF is a top oncogene among genes, the mutation of which is enriched in NR cells. The x-axis depicts the different types of mutations (e.g., amplification, deletion, missense) found per gene. Top hits are indicated. **(B)** Volcano plot showing the results of the Fisher’s exact test analysis for the mutational enrichment data shown in (A). Top hits are indicated. **(C)** BRAF-activating mutations (BRAF-MUT, e.g., V600E mutation) predict loss of RIPK3 expression in cancer. GDSC database was employed in the analysis. All BRAF-activating mutations were pooled into one group (BRAF-MUT). **(D)** Inhibition of BRAF in melanoma patients can rescue loss of RIPK3 expression. RIPK3 mRNA levels are increased in 58.3% of melanoma patient tumor biopsies following treatment with BRAF inhibitors Dabrafenib or Vemurafenib. Inset shows percentages of patients with significant changes in RIPK3 expression (Dataset GEO ID: GSE50509). **(E)** Inhibition of BRAF in cancer cell lines can rescue loss of RIPK3 expression. qRT-PCR and western blotting analysis of RIPK3 expression in ES2 and SkMel28 cell lines following 4 days of BRAF inhibition by 1 μM of TAK-632. These cell lines were selected because they did not show significant cell death following treatment with this inhibitor. The experiments were repeated two times. Bar graphs show means ± SEM with *t* test *p*-values. **(F)** Inhibition of BRAF in cancer cell lines can rescue loss of necroptosis sensitivity. A375 and SkMel28 cells were treated with indicated concentrations of Vemurafenib or TAK-632 for 4 days. Drugs were washed out and necroptosis was induced by 24-hour treatment with 25 ng/mL TNFα + 0.5 μM SM-164 + 30 μM zVAD.fmk. Cell survival was determined using CellTiterGlo assay. The underlying data can be found in S1 Data. BRAF-MUT, BRAF-activating mutation; BRAF-WT, BRAF wild-type; GDSC, Genomics of Drug Sensitivity in Cancer; NR, necroptosis-resistant; NS, necroptosis-sensitive; qRT-PCR, quantitative real-time PCR; TSZ, TNFα+SM-164+zVAD.fmk; UT, untreated

Using the differential sensitivity to necroptosis data from the cell-based screen, we performed a second round of bioinformatics analysis with a focus on genome-wide mutational enrichment in NR (fully resistant to necroptosis even at 1 μM of SM-164) versus necroptosis-sensitive (NS) cell lines. Our analysis revealed that several oncogenic mutations, including those of BRAF, are strongly enriched in the NR cell lines, compared to the NS cell lines (Fig 4A and 4B). Interestingly, 75 of the NR cell lines were found to have high RIPK3 expression (S6A Fig). Mutational enrichment analysis of the NR-RIPK3^high^ versus NR-RIPK3^low^ populations revealed 73 interesting genes, mutations of which may lead to necroptosis resistance via alternative pathways, independent of the RIPK3 expression suppression (S6B Fig).

Due to the importance of BRAF overactivation in cancer, we next focused on this oncogene. Transcriptomics analysis of the screened cell lines showed that mutations that lead to overactivation of BRAF can predict the loss of RIPK3 expression levels in cancer, despite many of the BRAF^WT^ cell lines displaying low RIPK3 expression, consistent with its heterogeneous nature (Fig 4C, S4 and S5 Tables), similar to that of high AXL expression levels.

These results raised the question of whether inhibition of BRAF, similar to that of AXL, could also result in reversal of the RIPK3 expression loss. Indeed, a transcriptomics study [57] analyzing melanoma patient tumor biopsies before and after treatments with BRAF inhibitors Dabrafenib and Vemurafenib revealed that RIPK3 expression was increased by at least 1.2-fold in 58.3% of the patients and decreased by least 1.2-fold in 25% of the patients, while no change was observed in 16.7% of the patients, consistent with the heterogeneous nature of RIPK3 expression loss (Fig 4D). Importantly, treatment of ES2 and SkMel28 cell lines, both of which carry an activating BRAF V600E mutation and have no initial RIPK3 expression, with low concentrations of BRAF inhibitor TAK-632 for 4 days resulted in an up-regulation of RIPK3 expression (Fig 4E). Importantly, this treatment also restored the sensitivity of these cells to TSZ-induced necroptosis (Fig 4F).

These findings suggest that oncogenic BRAF overactivation promotes the loss of RIPK3 expression and escape from necroptosis, which may be reversed upon inhibition of BRAF. Moreover, BRAF overactivating mutations are potential predictors/biomarkers for loss of RIPK3 expression and necroptosis resistance in cancer.

## Discussion

**Fig 5:**
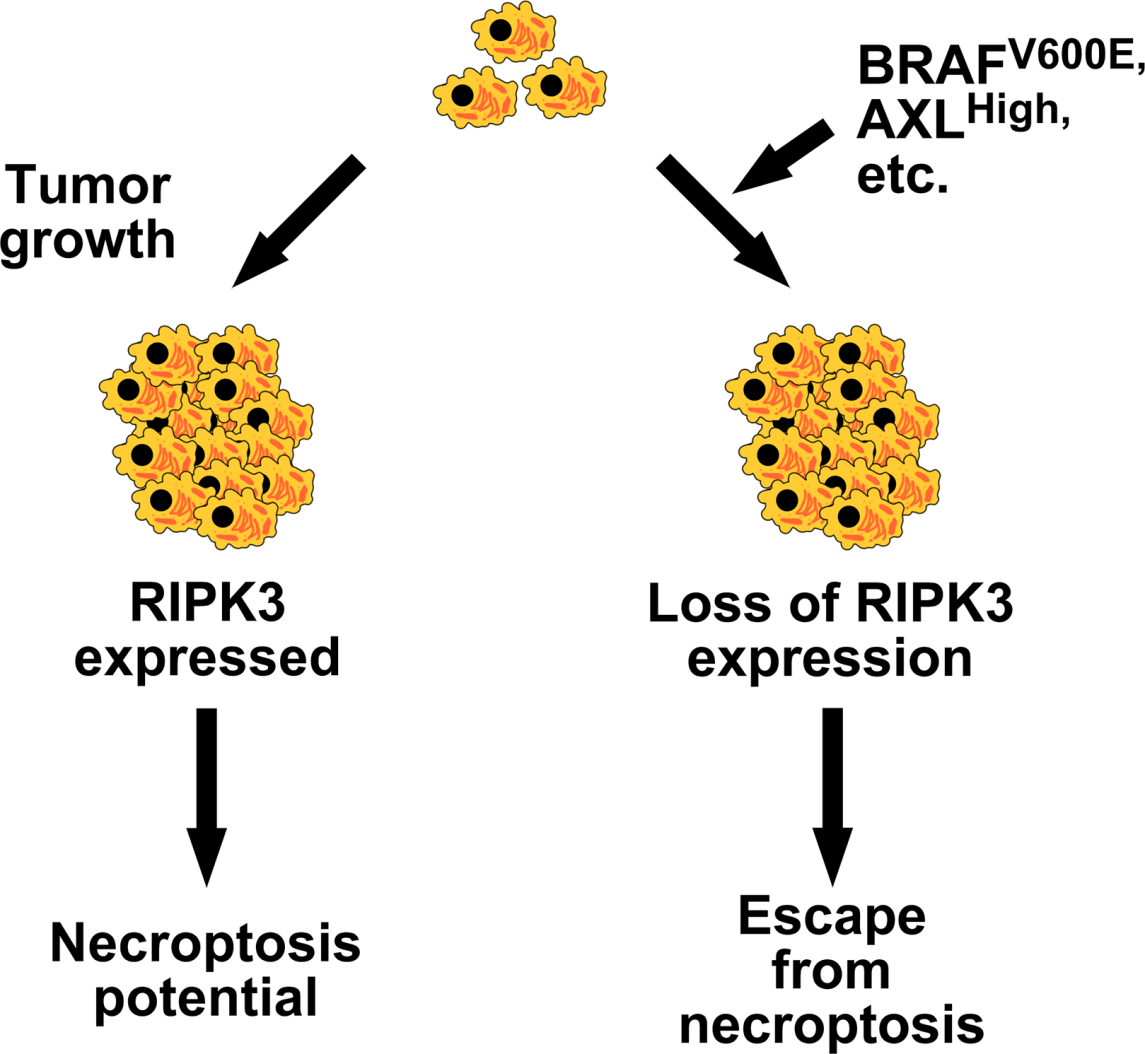
Role of oncogenes in the escape from necroptosis in cancer. Oncogenic BRAF (e.g., V600E mutation), AXL overexpression, and other oncogenic factors may promote escape from necroptosis via suppression of RIPK3 expression during tumorigenesis.

Here, we establish that necroptosis resistance can be found in high percentages of cancer cell lines derived from cancers of different tissue and cell type origins. We discover BRAF and AXL as the first two oncogenes that can drive the loss of RIPK3 expression in cancer cells (Fig 5). BRAF gain-of-function mutations and AXL overexpression, which are both observed in various cancers at high frequencies, are important therapeutic targets for the treatment of cancers. Interestingly, we found that the expression of RIPK3 may be restored upon inhibition of BRAF and AXL.

The loss of RIPK3 is a heterogeneous event, and its extent differs across various cancer cases, as can be seen from the screen data (Fig 2 and Fig 3) and the xenograft data (Fig 1 and S1A Fig). However, the prevalent loss of RIPK3 expression (Fig 3D) and resistance to necroptosis may be an important factor to consider during design of anticancer therapies. Our results suggest that therapies targeting key oncogenes BRAF and AXL result in a regain of RIPK3 expression in cancers that have lost it. Therefore, combinations of the compounds targeting these oncogenes with strategies that aim to induce necroptosis in tumors might augment the therapeutic benefit, because the regain of RIPK3 expression induced by the BRAF or AXL inhibitors is expected to render the tumors necroptosis sensitive.

However, because we show tumors undergo necroptosis in vivo, this inflammatory mode of cell death could positively contribute to tumor growth. Therefore, one needs to consider the potentially negative consequences of reactivating necroptosis by inducing the lost RIPK3 expression, because increase in necroptosis and inflammation can fuel tumor growth. On the other hand, RIPK3 has been shown to be important for CD8+ T-cell cross-priming and antitumor immunity [22]; therefore, inducing RIPK3 expression in tumor cells could increase their clearance by CD8+ T cells. Thus, induction of RIPK3 expression in cancer could prove to be a double-edged sword. Furthermore, while RIPK3-induced cytokine production and necroptosis-induced inflammation (or necroinflammation) [58] can fuel the tumor cell growth, such RIPK3-dependent processes may also promote antitumor immunity and programmed cell death of the tumor cells. It is conceivable that uncoupling necroptotic cell death, the pro-growth inflammation it brings, and CD8+ T-cell cross-priming induction could bring forward the benefits of RIPK3 expression induction in cancer (i.e., antitumor immunity stimulation via cross-priming) and diminish its disadvantages (inflammation, increased tumor growth and necrosis).

The presence of MLKL phospho-Ser358 marker in the tumor xenografts (Fig 1D) may also indicate that other roles of MLKL unrelated to cell death are at play during tumorigenesis, because phosphorylation of MLKL at this residue has been shown to be not sufficient to commit to necrotic cell death, as demonstrated in a recent study that links the ESCRT-III complex downstream of MLKL [59]. For instance, during tumorigenesis, MLKL/ESCRT-III pathway could be promoting CD8+ T-cell cross-priming and enhancing antitumor immunity, as ESCRT-III was found to be involved in cross-priming by necroptotic cells [59].

Investigating the role of the RIPK3 expression regain in cancer resistance and tumor regrowth in patients following BRAF inhibitor therapies (e.g., melanoma) could be of importance to explain this clinically vital phenomenon. We found that 38 out of 39 melanoma cell lines that have an activating BRAF mutation are fully resistant to necroptosis and have lost RIPK3 expression (S5 Table). Thus, according to our findings, RIPK3 expression is expected to be induced in most anti-melanoma therapies that employ mutant BRAF-specific inhibitors. It would be important to investigate if the regain of RIPK3 expression plays a role in the success or failure of BRAF-targeting therapies, in order to enhance the success rate and overcome the failures.

We analyzed the mutational status of BRAF and AXL kinases in the cell lines used for the aforementioned xenograft transcriptomics study. Consistent with the notion that oncogenic BRAF and AXL kinases promote the loss of RIPK3 expression in cancer cells, 14 out of 20 cell lines that harbor mutations promoting BRAF activation or high levels of AXL (or TYRO3) experienced loss of RIPK3 expression during in vivo passaging, while 13 out of 16 cell lines that lack such mutations did not experience that effect (S6 Table). The latter set of cells provides a crucial negative control and further supports that BRAF and AXL overactivation in cancer may drive the loss of RIPK3 during tumor progression.

It is possible that the selective pressure to lose RIPK3 expression during tumorigenesis comes from the necessity to evade immunity. For example, loss of RIPK3 in tumors would result in decreased cross-priming [22] and increased escape from immunity, thus benefiting tumor survival and growth, but inadvertently it would also result in loss of necroptosis potential because of the essentiality of RIPK3 for necroptosis. Thus, the cost/benefit for a tumor to lose RIPK3 expression could be dependent on the extent of necessity for the tumor cells to evade the immunity of the patient. This could explain why some cell lines obtained from the patients still had not lost RIPK3 expression but lose it when xenografted into mice.

Our in vivo results and published tumor xenograft experiments using immunocompromised animals show that the adaptive immune response (e.g., T cells) is not necessary for the loss of RIPK3 expression in tumor cells. Hence, our findings suggest that RIPK3 loss may be dependent on a tumor cell–intrinsic mechanism in vivo or due to interactions with stromal or innate immune cells.

RAS isoforms, known to be upstream of BRAF, were found among 20 oncogenes identified to positively correlate with resistance to necroptosis, further suggesting the involvement of BRAF in escape from necroptosis (S3A Fig). Cancer cell lines with BRAF mutations did not show as high correlation between AXL overexpression and RIPK3 as those with wild-type BRAF, suggesting that oncogenic pressure from either BRAF or AXL is sufficient to promote RIPK3 expression loss, and escape from necroptosis in cancer (S7 Fig). Overall, these observations strongly suggest that pathways downstream of BRAF and AXL are responsible for RIPK3 expression suppression and escape from necroptosis in cancer.

RIPK3 expression has been previously shown to be controlled via transcriptional repression mechanisms that include promoter hypermethylation and regulation via transcription factor Sp1 [21,60]. BRAF and AXL pathways are known to regulate many transcription factors, including JUN, FOS, ETS, and MYC. It is possible that the pathways overactivated upon mutational overactivation of BRAF/AXL converge on a set of transcription factors that control RIPK3 expression during tumorigenesis. Interestingly, BRAF overactivating mutations have been previously linked to promoter hypermethylation of various genes [61–63]. The delineation of the exact mechanistic details downstream of BRAF/AXL and upstream of transcription factors that control RIPK3 transcription is likely to be of importance to our understanding of cancer escape from necroptosis and will be elucidated in future studies.

Notably, both ABIN-1 and OPTN expression levels were found to strongly correlate with AXL expression across the analyzed 1,000 cell lines (S4A Fig). It is noteworthy that both of these ubiquitin-binding proteins have recently been linked to the regulation of RIPK1 activation in necroptosis [64,65]. Whether AXL regulates apoptosis and necroptosis via controlling expression of these ubiquitin chain adapters will be elucidated in future studies.

It is interesting that both BRAF and RIPK3 are in the same kinome branch, namely, in the tyrosine-kinase like (TKL) family of kinases [66]. Notably, several BRAF inhibitors have been reported to inhibit RIPK3 kinase activity, highlighting this similarity in the kinase domain structure [67]. Such structural similarity suggests a potential convergence and importance of the TKL family in the regulation of processes involving RIPK3, including necroptosis, cytokine production, and immunity. In fact, many of the members of the TKL family include regulators of these processes, such as RIPK1, RIPK2, RIPK3, MLKL, and TAK1 as well as IRAK and LRRK kinases [66].

In conclusion, we provide the first systematic evidence that most human cancer cell lines escape from necroptosis, independent of their tissue of origin or cancer type, and identify the first two oncogenic alterations upstream of the RIPK3 expression suppression. We show that BRAF and AXL oncogene overactivation in cancers is likely to be among the driving forces for the loss of RIPK3 during tumorigenesis and the consequent escape from necroptosis, as well as other RIPK3-driven processes. Understanding the mechanism of escape from necroptosis in tumorigenesis is likely to pave the way for development of better anticancer therapies.

## Materials and methods

### Reagents and antibodies

BMS-777607 and TAK-632 were purchased from SelleckChem (Houston, TX). Luminol (A8511), p-coumaric acid (C9008), Tween 20, and zVAD.fmk were from Sigma (St. Louis, MO). DMSO (sc-20258) was from Santa Cruz Biotechnology (Santa Cruz, CA). The following antibodies were used in this study: RIPK1 (Cell Signallng Technology [Danvers, MA], #3493); p-MLKL (S358) (Abcam (Cambridge, UK), ab187091); hMLKL (Abcam [Cambridge, UK], ab183770); and Actin (Santa Cruz Biotechnology (Santa Cruz, CA), sc-81178). Smac mimetic SM-164 was custom synthesized (SelleckChem [Houston, TX]) [7]. TNFα was from Cell Sciences (Newburyport, MA).

### Cell lines

All cell lines were grown in RPMI or DMEM medium (Corning, with L-glutamine, with 4.5 g/L glucose, without pyruvate) supplemented with 10% FBS (Sigma), 1× penicillin/streptomycin (Life Technologies), 1 μg/mL amphotericin B (Santa Cruz Biotechnology, sc-202462A), 1× non-essential amino acids mix (NEAA MEM) (Gibco, Life Technologies) and 1 mM sodium pyruvate (Gibco, Life Technologies).

### Drug screen across large cell line collection

High-throughput drug screening and sensitivity modeling (curve fitting and IC_50_ estimation) was performed essentially as described previously [53]. Cells were cultured in RPMI or DMEM/F12 containing 5% FBS and penicillin/streptomycin. Cells were incubated at 37°C in a humidified atmosphere with 5% CO_2_. Cells were grown in RPMI or DMEM/F12 in order to minimize the potential effect of different cell culture media on the drug sensitivity during the screening. A panel of 92 SNPs was profiled for each cell line (Sequenom, San Diego, CA), in order to authenticate the cell lines and thus rule out cross-contamination. A pairwise comparison score was calculated for this purpose. Moreover, short tandem repeat (STR) analysis (AmpFlSTR Identifiler, Applied Biosystems, Carlsbad, CA) was done on the cell lines and the results were matched to existing STR signatures from the repository that provided the cell lines. Briefly, cells were seeded in 384-well plates at variable density to ensure optimal proliferation during the assay. Drugs were added to the cells the day after seeding for adherent cell lines and the day of seeding for suspension cell lines. For tumor subtypes containing both adherent and suspension cells, all lines were drugged the same day (small cell lung cancer cell lines, for example, were all drugged the day after seeding). A series of nine doses was used using a 2-fold dilution factor for a total concentration range of 256-fold. Maximum concentration was chosen for each drug based on prior knowledge of activity on target and in cells. Viability was determined using resazurin after 5 days of drug exposure. Cell lines were treated with TSZ: TNFα (fixed dose 20 ng/mL) + ZVAD (fixed dose 20 μM) + Variable dose of SM-164 (Max of 1.024 μM).

### Immunoblotting

Total cell lysates (20–30 μg) were heated at 90° for 5 minutes in 1× SDS-PAGE sample buffer (2% SDS, 1% beta-mercaptoethanol, 0.01% bromophenol blue, 50% glycerol, 63 mM Tris-HCl, pH 6.8), subjected to 10% SDS-PAGE using Bio-Rad’s Mini-PROTEAN Electrophoresis System, and then electrotransferred onto 0.2-μm nitrocellulose membranes (buffer: 5.82 g/L Tris, 2.93 g/L glycine, 20% ethanol) for 2 hours at 0.4 A current, with the wet transfer tank submerged into an ice/water bath using Bio-Rad’s Trans-Blot cell. Membranes were blocked for 1 hour in TBST buffer containing 5% (w/v) nonfat milk and probed with the indicated antibodies in TBST containing 5% (w/v) BSA for 16 hours at 4°. Detection was performed using HRP-conjugated secondary antibodies and in-house-made chemiluminescence reagent (2.5 mM luminol, 0.4 mM p-coumaric acid, 100 mM Tris-HCl, pH 8.6, 0.018% H_2_O_2_).

### RIPK3 expression and necroptosis sensitivity regain experiments

Cells were seeded into 24-well plates in 1 mL of medium at 15%–20% confluence. Cells were treated 16–24 hours later with BMS-777607, TAK-632, or Vemurafenib (0.3–3 μM) for 96 hours. Cells were washed twice with 1 mL of medium (5-minute incubation at 37° for each wash) and pretreated with 0.5 μM SM-164 and 30 μM zVAD.fmk for 30 min with a subsequent treatment with 25 ng/mL hTNFα for 24 hours to induce necroptosis. Cell survival was determined using CellTiterGlo (Promega) kit according to manufacturer’s instructions. Equal volumes of the reagent were added to the culture medium and the 24-well plates were incubated at 25° for 10 minutes in the dark, with agitation. A total of 25 μL of the obtained lysates were transferred into opaque 384-well plates and luminescence was measured at 100 sensitivity setting with 0.2 seconds integration time, using BioTek Synergy 2 plate reader. For RIPK3 expression analysis, cells were lysed in RLT buffer of the RNeasy kit (Qiagen).

### qRT-PCR

RNA was isolated using RNeasy kit (Qiagen) and cDNA synthesis was performed using RNA to cDNA EcoDry Premix (Double Primed) (Takara Bio). A total of 1 μg of RNA was used per premix tube. Quantitative real-time PCR (qRT-PCR) was done using SYBR Green Real-Time PCR Master Mix (Thermo Fisher Scientific), with QuantStudio 7 Flex Real-Time PCR System (Thermo Fisher Scientific). RIPK3 qRT primer sequences (hRIPK3_F, CAAGGAGGGACAGAAATGGA; hRIPK3_R, GCCTTCTTGCGAACCTACTG) were as described elsewhere [21].

### Mouse xenograft experiments

Experiments were performed as previously described [68]. Tumor ascites from patients with advanced ovarian cancer (IRB approved protocols at Dana-Farber Cancer Institute) were implanted orthotopically (intraperitoneal injection) in NOD-SCID mice (8 weeks old, Jackson labs). Mice were followed weekly for abdominal distension and were humanely killed 3–8 months after injection of the original patient tumor ascites (passage 0) to harvest tumor ascites for serial passaging. Ascites harvested from the xenografts were processed for red blood cell lysis and serially passaged (up to 3 passages) in new NOD-SCID mice. Tumors were frozen in liquid nitrogen for storage. Tumors were lysed in NP-40 lysis buffer (25 mM HEPES [pH 7.5], 0.2% NP-40, 120 mM NaCl, 0.27 M sucrose, 5 mM EDTA, 5 mM EGTA, 50 mM NaF, 10 mM b-glycerophosphate, 5 mM sodium pyrophosphate, 1 mM Na_3_VO_4_ (fresh), 1 mM benzamidine [fresh], 0.1% BME [fresh], 1 mM PMSF [fresh], 2× Complete protease inhibitor cocktail [Roche]) using VWR 200 Homogenizer, on ice. Lysates were cleared by centrifugation at 16,000*g*, 15 minutes, 4°. Protein concentrations were determined using Bradford reagent (BioRad). Protein samples were mixed with 5× SDS-PAGE sample buffer and frozen at −80° for storage.

### Statistics and bioinformatics

For all experiments, unless otherwise indicated, *n* was at least 3. Statistical analyses were performed using GraphPad Prism 7 or Microsoft Excel. Violin and bean plots were made using BoxPlotR (http://shiny.chemgrid.org/boxplotr/) [69]. Data were analyzed using one-way analysis of variance (ANOVA) test with Bonferroni posttest for non-paired datasets. Student *t* test was used for paired datasets. Data points are shown as means ± SEM. ClustVis was used for heatmap generation [70]. The heatmap in Fig 2D was generated as follows. The data IC_50_ values from the screen and gene expression values from GCSD database were analyzed by z-test and the heatmap was generated from these z-scores. ClustVis Data Pre-Processing settings were as follows: no row centering, unit variance scaling. Column settings were as follows: clustering distance—Manhattan; clustering method—single; tree ordering—original. Row settings were as follows: no clustering.

The following databases were used for bioinformatics analysis of published datasets: cBio Cancer Genomics Portal (http://www.cbioportal.org/) [51], Broad-Novartis Cancer Cell Line Encyclopedia [55] (http://www.broadinstitute.org/ccle/home, CCLE_Expression_Entrez_2012-10-18.res microarray dataset), Genomics of Drug Sensitivity in Cancer [54] (http://www.cancerrxgene.org) and Gene Expression Omnibus (http://www.ncbi.nlm.nih.gov/geo/).

The following datasets were included in this manuscript: GDS5336 [46], GDS4367 [47], GDS3894 [48], GDS2546 [49], microarray datasets from Ma and colleagues [50], and GSE48433 [52] (see S7 Table).

The oncogene-related gene database was obtained by searching Uniprot database for key word “oncogene” (QUERY: keyword:oncogene AND organism:"Homo sapiens (Human) [9606]"). Intersections of gene lists were made with CrossCheck [71] and Venny (http://bioinfogp.cnb.csic.es/tools/venny/).

Mutational enrichment was done by dividing the number of mutations identified in NR cells by those identified in cells that were sensitive to the necroptosis treatment (NS) and then performing a z-test on this dataset. NR cells were defined as those that had no reduction in cell viability at 1 μM SM-164 concentration (the highest concentration used in the screen). The rest of the cell lines that exhibited reduction in cell viability were defined as NS.

## Supporting information

S1 Data Excel file containing the underlying numerical data for Figs 1A, 1B, 1C, 1E, 1F, 2B, 2E, 2F, 3A, 3B, 3C, 3D, 3E, 3F, 4A, 4B, 4C, 4D, 4E, 4F, S1B, S1C, S2A, S2B, S2C, S3B, S4B, S5A, S5B, S5C, S5D, S6A, S6B and S7. Data are given in indicated separate sheets.
(XLSX)

S1 Fig **(A)** Loss of RIPK3 expression during tumorigenesis. RIPK3 expression is progressively lost during tumorigenesis. Ovarian PDX lysates obtained at the indicated in vivo passages were immunoblotted with the indicated antibodies. The lack of RIPK3 expression loss in all of the PDX samples highlights the heterogeneity of this event in cancer. **(B)** Effect of Nec-1 and GSK’872 on cell death induced by TSZ. The experiment shown in Fig 1F was repeated using indicated TNFα, SM-164, and zVAD.fmk concentrations and the effects of the RIPK1 inhibitor Nec-1 and the RIPK3 inhibitor GSK’872 on cell death were tested at the indicated concentrations. Cell death was assessed using Toxilight assay at 4 hours. **(C)** As in (B), except indicated doses and the MLKL inhibitor NSA were used. The underlying data can be found in S1 Data. NSA, necrosulfonamide; PDX, patient-derived xenograft; TSZ, TNFα+SM-164+zVAD.fmk
(TIF)

S2 Fig Necroptosis sensitivity screen confirmation by TCZ treatment and distribution of the cell lines in the screen across tissue types. **(A)** Low-throughput confirmation of the screen observations regarding necroptosis resistance. Indicated cells were treated with TCZ (TNFα = 20 ng/mL; CHX = 0.5 μg/mL, 30-minute pretreatment; zVAD = 25 μM, 30-minute pretreatment) ± Nec-1 indicated treatments and cell survival was measured 16 hours later using CellTiterGlo. Means ± SEM are shown with *t* test *p*-values. **(B)** Frequency of necroptosis-resistant cancer cell lines across various tissues of origin. Some tissues did not have NS cancer cell lines (e.g., brain, testes, and uterus), while only 40% of cancer cell lines from the biliary tract were resistant to necroptosis. **(C)** Numbers of cancer cell lines used in the screen across various tissues of origin. A total of 91 cell lines were derived from leukemia patients and 102 cell lines were from NSCLC. The underlying data can be found in S1 Data. CHX, Cycloheximide; NSCLC, non-small-cell lung carcinoma; TCZ, TNFα+Cycloheximide+zVAD.fmk
(TIF)

S3 Fig High-throughput screening data analysis strategy and enrichment of low RIPK3 expression levels in necroptosis-resistant cell lines. **(A)** Outline of the data analysis strategy for differential necroptosis sensitivity data from the cell-based high-throughput screen described in Fig 1 that identified 20 oncogene-related genes that correlate with high necroptosis resistance and low RIPK3 expression. **(B)** Low RIPK3 expression levels are enriched in cancer cell lines fully resistant to necroptosis. The GDSC database was employed for the analysis. Means, 10–90 percentile data points ± SEM are shown with *t* test *p*-values. The underlying data can be found in S1 Data. GDSC, Genomics of Drug Sensitivity in Cancer.
(TIF)

S4 Fig AXL/TYRO3 expression correlation with expression of RIPK3 versus other necroptosis-related genes. **(A)** Pearson correlation analysis using CCLE mRNA expression database for known necroptosis-related proteins was performed and a heatmap for the Pearson coefficients was generated using ClustVis. **(B)** High AXL expression positively correlates with low RIPK3 expression levels in SAC (TCGA, Nature 2014 dataset) and AML (TCGA, Provisional dataset), according to Pearson and Spearman correlation analyses. Cell lines with either genomic AXL or RIPK3 mutations were omitted from the analysis (18 for SAC and 0 for AML). The results shown here are based upon data generated by the TCGA Research Network, using cBioportal. The underlying data can be found in S1 Data. AML, acute myeloid leukemia; CCLE, Cancer Cell Line Encyclopedia; SAC, stomach adenocarcinoma; TCGA, The Cancer Genome Atlas.
(TIF)

S5 Fig AXL/TYRO3 expression correlation with expression of RIPK3 and TSZ-IC_50_ values. **(A)** High AXL expression positively correlates with resistance to TSZ-induced necroptosis. Pearson correlation analysis for AXL mRNA levels versus TSZ-IC_50_ values for the screened cancer cell lines. Markers stacked at IC_50_ = 1 value indicate cell lines with no response to TSZ even at the highest SM-164 concentration of 1 μM. The CCLE database was employed for the analysis. **(B)** High AXL expression positively correlates with low RIPK3 expression levels. Pearson and Spearman correlation analyses were used. The CCLE database was employed for the analysis. **(C)** High AXL expression positively correlates with low RIPK3 expression levels. The GDSC database was employed for the analysis. Quartile analysis using one-way ANOVA was used to determine statistical significance. **(D)** High TYRO3 expression positively correlates with low RIPK3 expression levels. The GDSC database was employed for the analysis. Quartile analysis using one-way ANOVA was used to determine statistical significance. The underlying data can be found in S1 Data. CCLE, Cancer Cell Line Encyclopedia; GDSC, Genomics of Drug Sensitivity in Cancer.
(TIF)

S6 Fig Mutational enrichment analysis for NR-RIPK3^high^ cell lines. **(A)** A total of 75 of the NR cell lines have not lost RIPK3 expression (RIPK3^high^). RIPK3^high^ subpopulation was defined as cell lines with RIPK3 expression greater than the third quartile of the 941-cell-line population. NR subpopulation was defined as cell lines that showed no cell death at the highest SM-164 concentration (1 μM). **(B)** Fold enrichment of mutations in NR-RIPK3^high^ versus NR-RIPK3^low^ cell lines is plotted against the genes and mutation types. All the displayed hits pass the Fisher’s exact test with *p* < 0.05 for mutational enrichment in the NR-RIPK3^high^ population. Types of mutations are indicated. The underlying data can be found in S1 Data. AMP, amplification; DEL, deletion; MUT, point mutation; NR, necroptosis-resistant;
(TIF)

S7 Fig High AXL expression positively correlates with low RIPK3 expression levels in cell lines with wild-type BRAF, and this correlation is decreased in cell lines with mutant BRAF. Pearson *p*-values were used for the analysis. The underlying data can be found in S1 Data.
(TIF)

S1 Table (DOCX)

S2 Table (DOCX)

S3 Table (DOCX)

S4 Table (DOCX)

S5 Table (DOCX)

S6 Table (DOCX)

S7 Table (DOCX)

## Abbreviations

CCLE: Cancer Cell Line Encyclopedia
GDSC: Genomics of Drug Sensitivity in Cancer
TCGA: The Cancer Genome Atlas
IAP: inhibitor of apoptosis protein
MLKL: Mixed lineage kinase domain-like protein
NEAA: non-essential amino acids
NR: necroptosis-resistant
NS: necroptosis-sensitive
NSA: necrosulfonamide
PDX: patient-derived xenograft
qRT-PCR: quantitative real-time PCR
RIPK1: Receptor-interacting serine/threonine-protein kinase 1
RIPK3: Receptor-interacting serine/threonine-protein kinase 3
STR: short tandem repeat
TAM: Tyro3, Axl, Mer
TKL: tyrosine-kinase like
TCZ: TNFα+Cycloheximide+zVAD.fmk
TNFα: Tumor necrosis factor alpha
TSZ: TNFα+SM-164+zVAD.fmk
