# Supplementary figures and images for: BRAF and AXL oncogenes drive RIPK3 expression loss in cancer
Source: PLoS Biol. 2018 Aug 29;16(8):e2005756. doi: 10.1371/journal.pbio.2005756 (PMC6114281; doi:10.1371/journal.pbio.2005756)

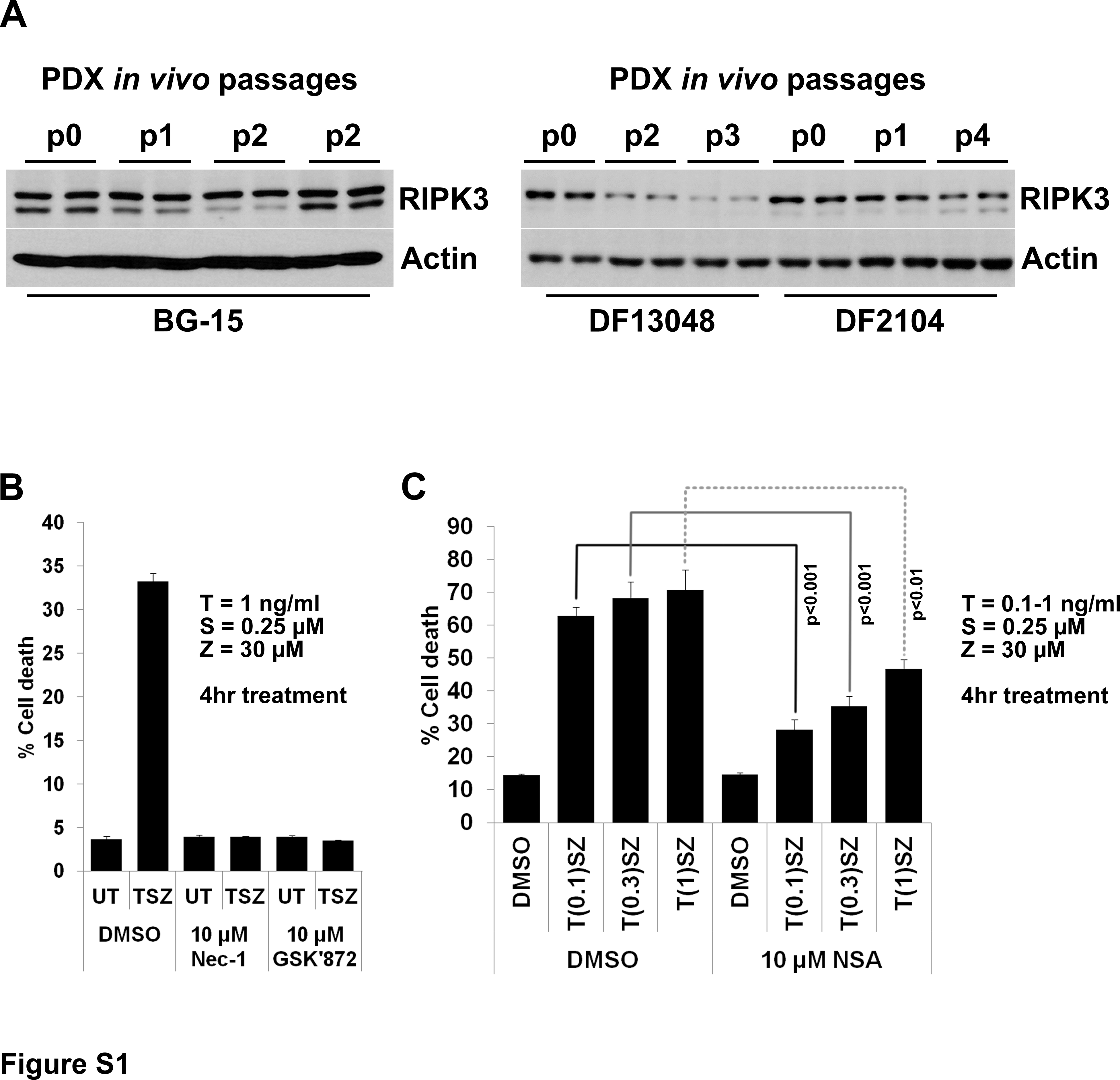

Supplement: S1 Fig — (A) Loss of RIPK3 expression during tumorigenesis. RIPK3 expression is progressively lost during tumorigenesis. Ovarian PDX lysates obtained at the indicated in vivo passages were immunoblotted with the indicated antibodies. The lack of RIPK3 expression loss in all of the PDX samples highlights the heterogeneity of this event in cancer. (B) Effect of Nec-1 and GSK’872 on cell death induced by TSZ. The experiment shown in Fig 1F was repeated using indicated TNFα, SM-164, and zVAD.fmk concentrations and the effects of the RIPK1 inhibitor Nec-1 and the RIPK3 inhibitor GSK’872 on cell death were tested at the indicated concentrations. Cell death was assessed using Toxilight assay at 4 hours. (C) As in (B), except indicated doses and the MLKL inhibitor NSA were used. The underlying data can be found in S1 Data. NSA, necrosulfonamide; PDX, patient-derived xenograft; TSZ, TNFα+SM-164+zVAD.fmk (TIF) [file pbio.2005756.s002.tif]

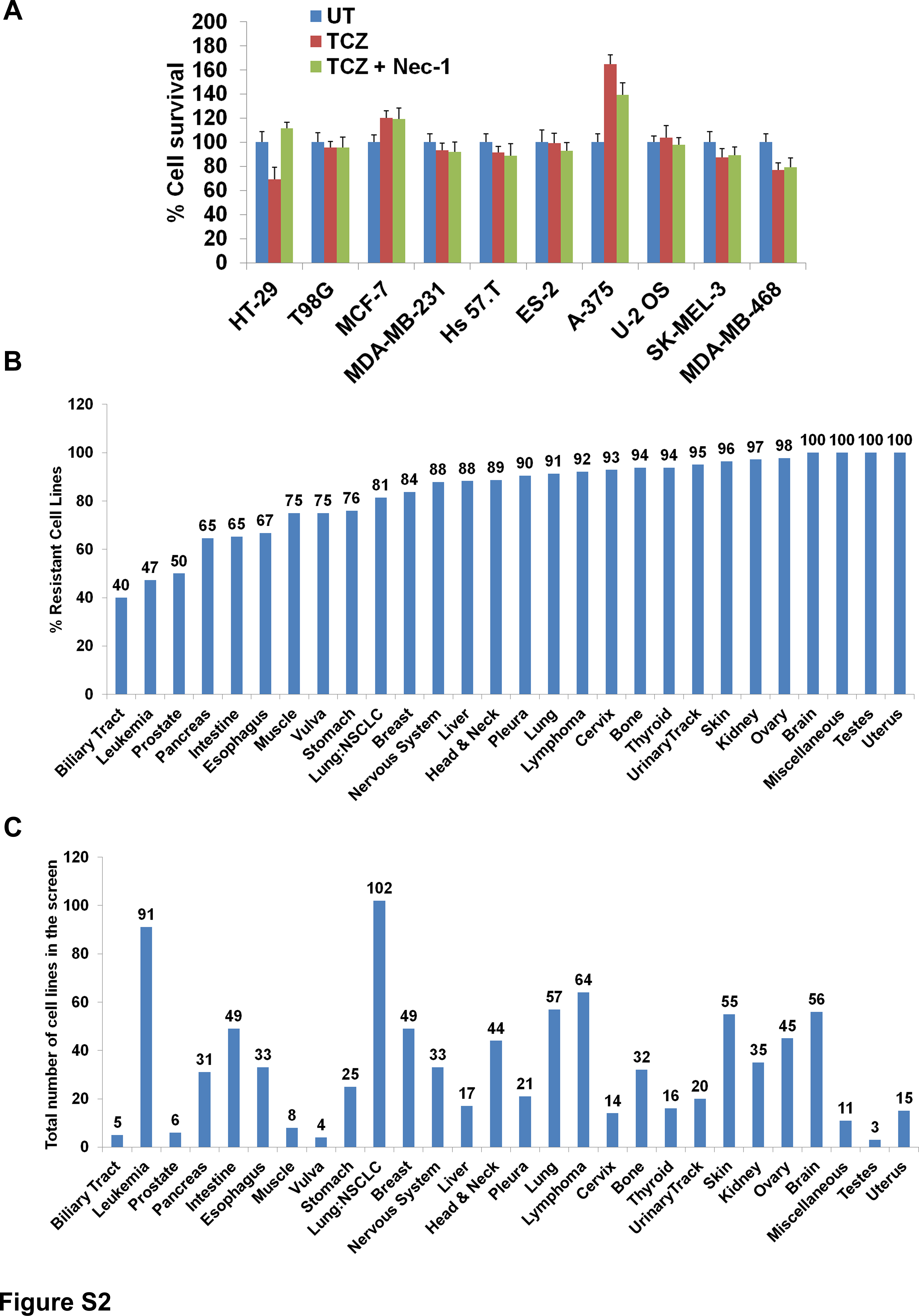

Supplement: S2 Fig — (A) Low-throughput confirmation of the screen observations regarding necroptosis resistance. Indicated cells were treated with TCZ (TNFα = 20 ng/mL; CHX = 0.5 μg/mL, 30-minute pretreatment; zVAD = 25 μM, 30-minute pretreatment) ± Nec-1 indicated treatments and cell survival was measured 16 hours later using CellTiterGlo. Means ± SEM are shown with t test p-values. (B) Frequency of necroptosis-resistant cancer cell lines across various tissues of origin. Some tissues did not have NS cancer cell lines (e.g., brain, testes, and uterus), while only 40% of cancer cell lines from the biliary tract were resistant to necroptosis. (C) Numbers of cancer cell lines used in the screen across various tissues of origin. A total of 91 cell lines were derived from leukemia patients and 102 cell lines were from NSCLC. The underlying data can be found in S1 Data. CHX, Cycloheximide; NSCLC, non-small-cell lung carcinoma; TCZ, TNFα+Cycloheximide+zVAD.fmk (TIF) [file pbio.2005756.s003.tif]

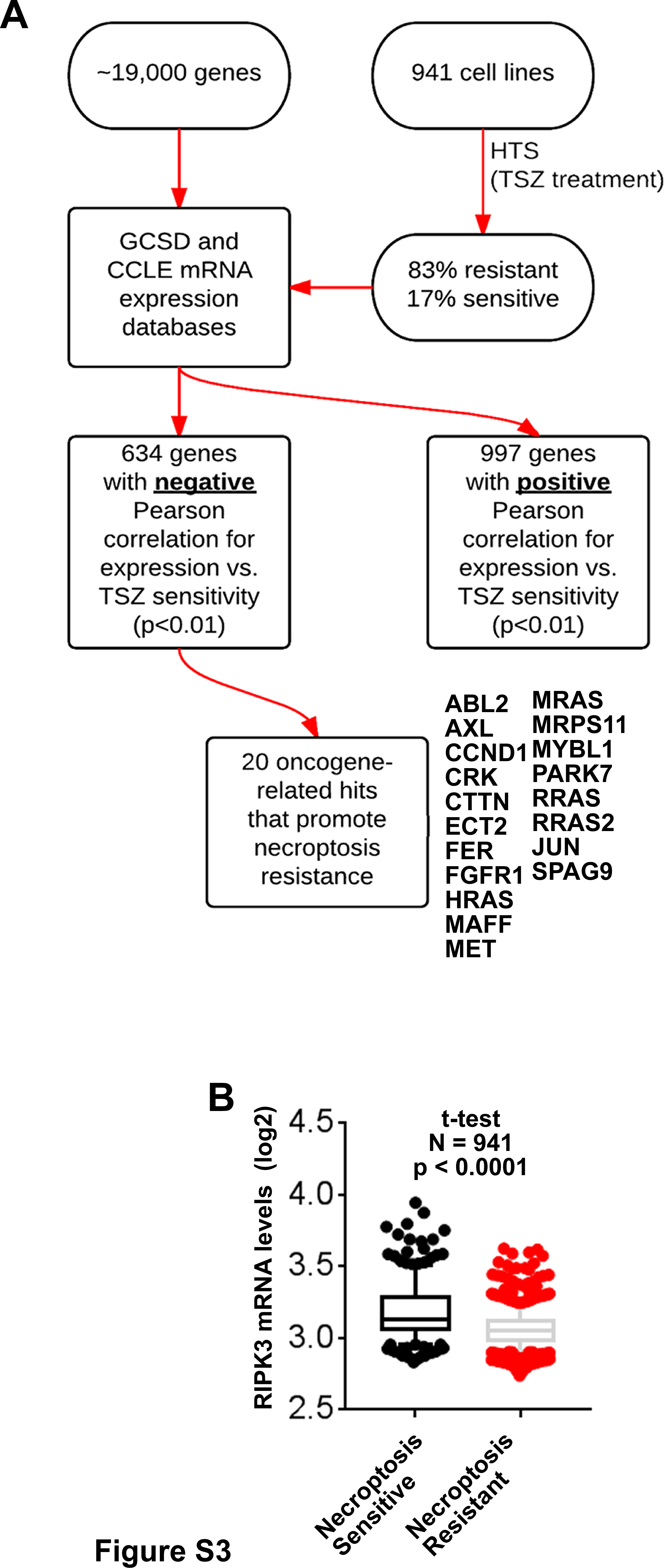

Supplement: S3 Fig — (A) Outline of the data analysis strategy for differential necroptosis sensitivity data from the cell-based high-throughput screen described in Fig 1 that identified 20 oncogene-related genes that correlate with high necroptosis resistance and low RIPK3 expression. (B) Low RIPK3 expression levels are enriched in cancer cell lines fully resistant to necroptosis. The GDSC database was employed for the analysis. Means, 10–90 percentile data points ± SEM are shown with t test p-values. The underlying data can be found in S1 Data. GDSC, Genomics of Drug Sensitivity in Cancer. (TIF) [file pbio.2005756.s004.tif]

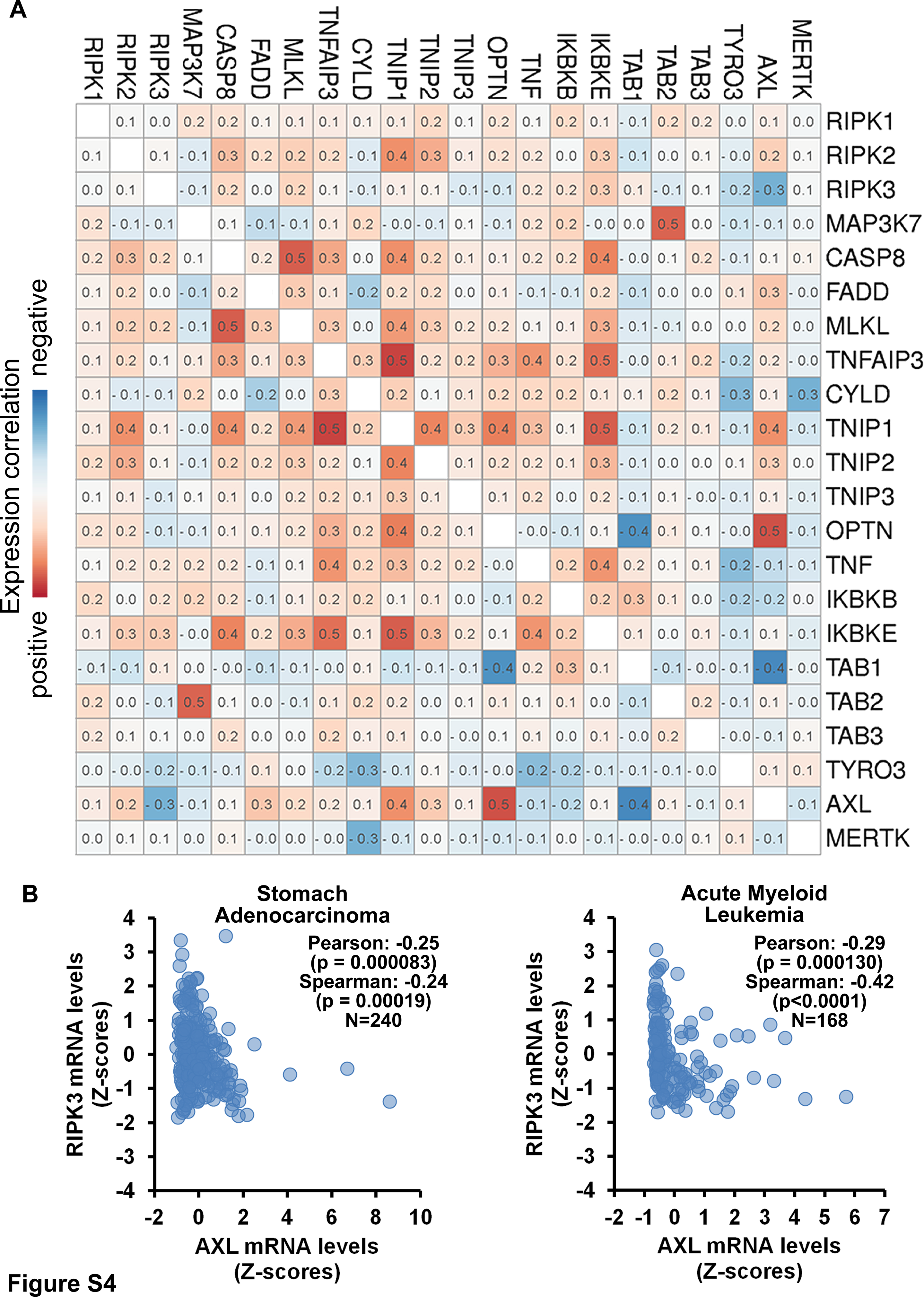

Supplement: S4 Fig — (A) Pearson correlation analysis using CCLE mRNA expression database for known necroptosis-related proteins was performed and a heatmap for the Pearson coefficients was generated using ClustVis. (B) High AXL expression positively correlates with low RIPK3 expression levels in SAC (TCGA, Nature 2014 dataset) and AML (TCGA, Provisional dataset), according to Pearson and Spearman correlation analyses. Cell lines with either genomic AXL or RIPK3 mutations were omitted from the analysis (18 for SAC and 0 for AML). The results shown here are based upon data generated by the TCGA Research Network, using cBioportal. The underlying data can be found in S1 Data. AML, acute myeloid leukemia; CCLE, Cancer Cell Line Encyclopedia; SAC, stomach adenocarcinoma; TCGA, The Cancer Genome Atlas. (TIF) [file pbio.2005756.s005.tif]

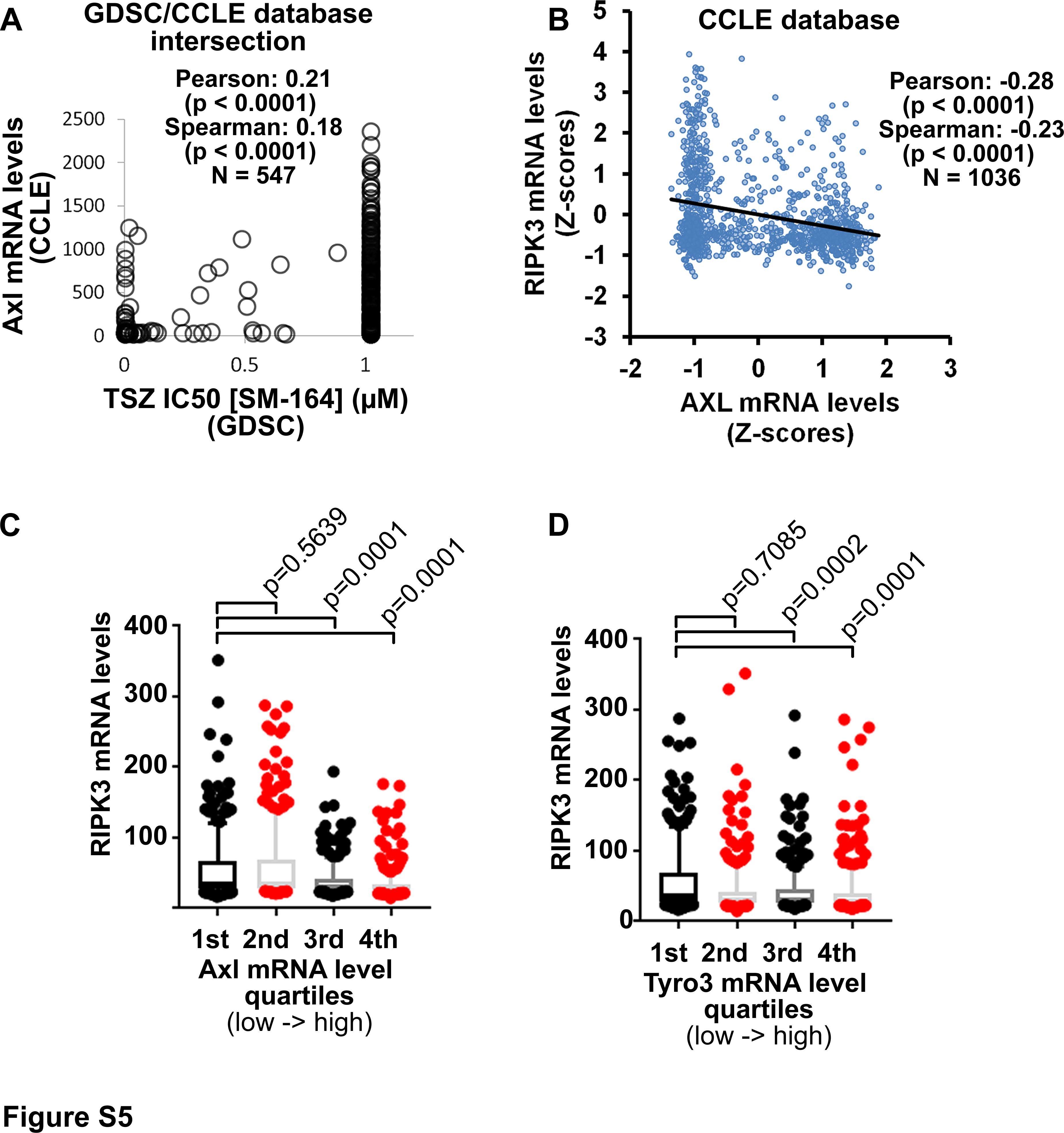

Supplement: S5 Fig — (A) High AXL expression positively correlates with resistance to TSZ-induced necroptosis. Pearson correlation analysis for AXL mRNA levels versus TSZ-IC50 values for the screened cancer cell lines. Markers stacked at IC50 = 1 value indicate cell lines with no response to TSZ even at the highest SM-164 concentration of 1 μM. The CCLE database was employed for the analysis. (B) High AXL expression positively correlates with low RIPK3 expression levels. Pearson and Spearman correlation analyses were used. The CCLE database was employed for the analysis. (C) High AXL expression positively correlates with low RIPK3 expression levels. The GDSC database was employed for the analysis. Quartile analysis using one-way ANOVA was used to determine statistical significance. (D) High TYRO3 expression positively correlates with low RIPK3 expression levels. The GDSC database was employed for the analysis. Quartile analysis using one-way ANOVA was used to determine statistical significance. The underlying data can be found in S1 Data. CCLE, Cancer Cell Line Encyclopedia; GDSC, Genomics of Drug Sensitivity in Cancer. (TIF) [file pbio.2005756.s006.tif]

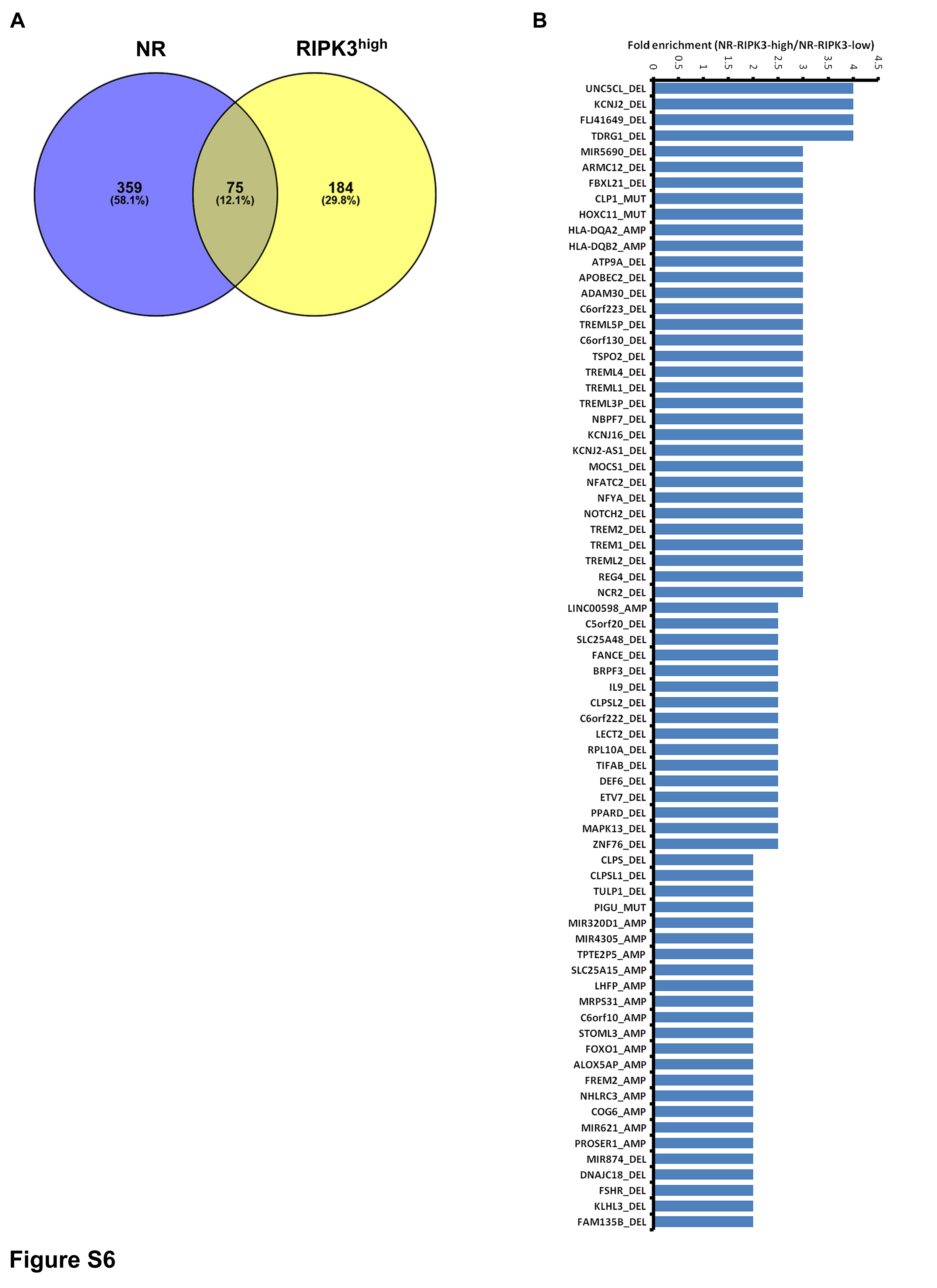

Supplement: S6 Fig — (A) A total of 75 of the NR cell lines have not lost RIPK3 expression (RIPK3high). RIPK3high subpopulation was defined as cell lines with RIPK3 expression greater than the third quartile of the 941-cell-line population. NR subpopulation was defined as cell lines that showed no cell death at the highest SM-164 concentration (1 μM). (B) Fold enrichment of mutations in NR-RIPK3high versus NR-RIPK3low cell lines is plotted against the genes and mutation types. All the displayed hits pass the Fisher’s exact test with p < 0.05 for mutational enrichment in the NR-RIPK3high population. Types of mutations are indicated. The underlying data can be found in S1 Data. AMP, amplification; DEL, deletion; MUT, point mutation; NR, necroptosis-resistant; (TIF) [file pbio.2005756.s007.tif]

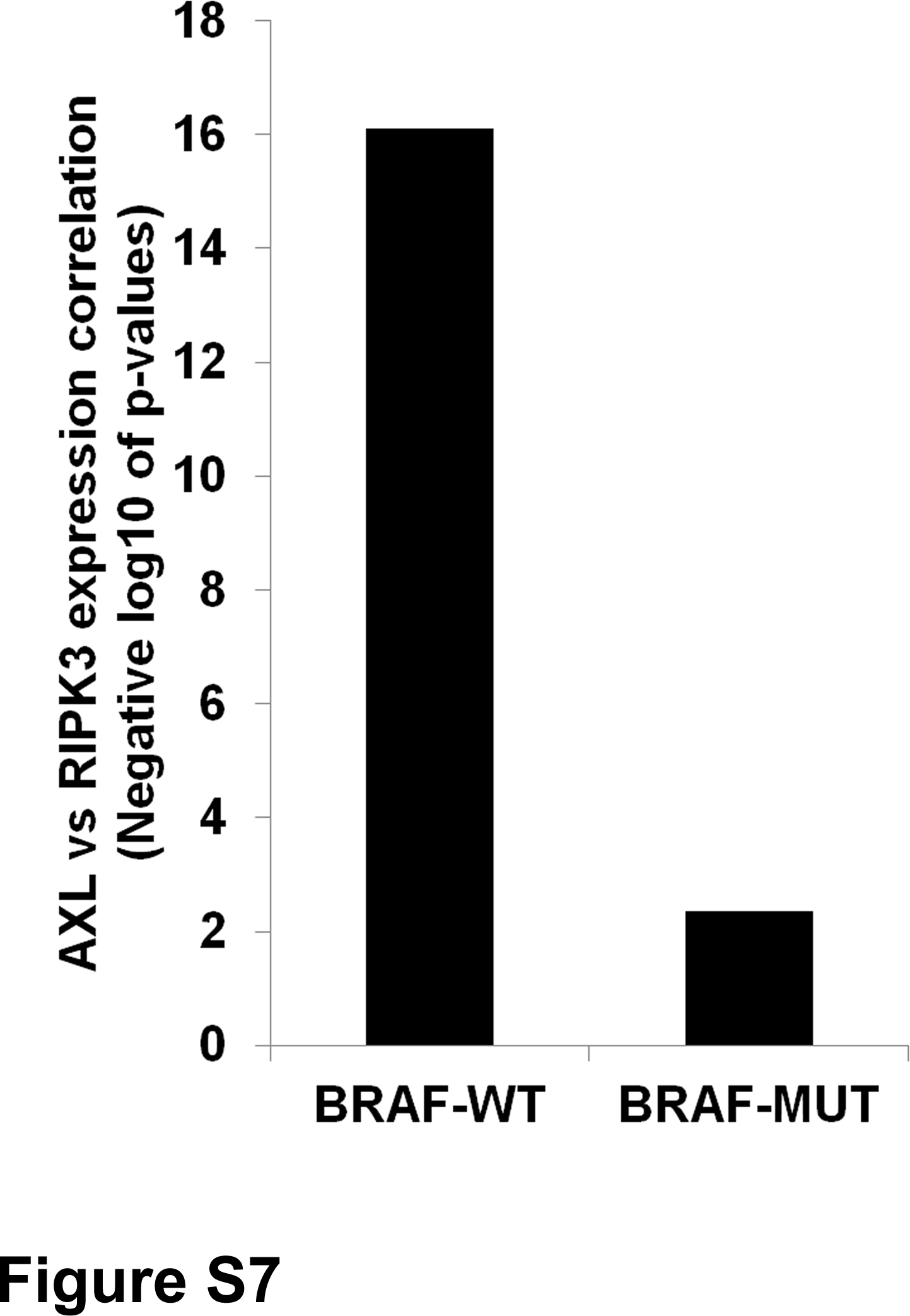

Supplement: S7 Fig — Pearson p-values were used for the analysis. The underlying data can be found in S1 Data. (TIF) [file pbio.2005756.s008.tif]
