# Supplementary material for: BRAF and AXL oncogenes drive RIPK3 expression loss in cancer
Source: PLoS Biol. 2018 Aug 29;16(8):e2005756. doi: 10.1371/journal.pbio.2005756 (PMC6114281; doi:10.1371/journal.pbio.2005756)
Supplement: S1 Table — (DOCX) [file pbio.2005756.s009.docx]

S1 Table. Cancer cell lines fully resistant to TSZ-induced necroptosis. No death was observed in these cell lines after TSZ treatment (T = 20 ng/ml TNFα, S = 1 µM SM-164, Z = 20 µM zVAD).

| **Biliary Tract** | CAS-1 | SF539 | MDA-MB-175-VII | KYSE-70 | PCI-4B |
| --- | --- | --- | --- | --- | --- |
| TGBC1TKB | CCF-STTG1 | SK-MG-1 | MDA-MB-231 | OACM5-1 | PCI-6A |
| TGBC24TKB | D-247MG | SNB75 | MDA-MB-330 | OE33 | PE/CA-PJ15 |
| **Bone** | D-263MG | SW 1088 | MDA-MB-361 | SK-GT-4 | RPMI 2650 |
| CADO-ES1 | D283 Med | SW 1783 | MDA-MB-436 | TE-1 | SACC-83 |
| CAL-72 | D-336MG | T98G | MDA-MB-453 | TE-10 | SAS |
| CAL-78 | D-392MG | U-118 MG | MFM-223 | TE-11 | SAT |
| CHSA8926 | D-423MG | U-251 MG | MRK-nu-1 | TE-5 | SCC-15 |
| CS1 | D-502MG | U-87 MG | T47D | TE-6 | SCC-9 |
| ES1 | D-542MG | YH-13 | UACC-812 | TE-8 | SKN-3 |
| ES3 | D-566MG | YKG-1 | UACC-893 | **Head & Neck** | **Intestine** |
| ES6 | Daoy | **Breast** | YMB-1-E | A253 | C2BBe1 |
| ES7 | DBTRG-05MG | AU565 | ZR-75-30 | ACC3 | CaR-1 |
| ES8 | DK-MG | BT-474 | **Cervix** | BB30-HNC | COLO 741 |
| EW-1 | GAMG | BT-549 | C-33 A | BB49-HNC | COLO-320-HSR |
| EW-16 | GB-1 | CAL-120 | Ca Ski | BHY | COLO-678 |
| EW-18 | GI-1 | CAL-148 | DoTc2 4510 | BICR 10 | HCC2998 |
| EW-22 | GMS-10 | CAL-51 | HeLa | BICR 31 | HCT 116 |
| EW-24 | H4 | CAL-85-1 | HT-3 | BICR 78 | HCT-15 |
| EW-7 | Hs 683 | COLO-824 | ME-180 | Ca9-22 | HT115 |
| G-292 | KALS-1 | DU4475 | MS751 | CAL 27 | HT55 |
| H-EMC-SS | KINGS-1 | EFM-19 | OMC-1 | CAL-33 | HUTU-80 |
| HOS | KNS-42 | EFM-192A | SiHa | Detroit 562 | KM12 |
| HuO-3N1 | KNS-81-FD | EVSA-T | SISO | FaDu | LoVo |
| HuO9 | KS-1 | HCC1143 | SKG-IIIa | H3118 | LS-123 |
| MG-63 | LN-18 | HCC1187 | SW756 | HCE-T | LS-411N |
| MHH-ES-1 | LN-229 | HCC1395 | TC-YIK | HN | MDST8 |
| NY | LN-405 | HCC1419 | **Esophagus** | HO-1-u-1 | NCI-H630 |
| Saos-2 | LNZTA3WT4 | HCC1428 | COLO-680N | HSC-2 | NCI-H716 |
| SJSA-1 | M059J | HCC1500 | EC-GI-10 | HSC-4 | NCI-H747 |
| SK-ES-1 | MOG-G-CCM | HCC1569 | HCE7 | JHU-011 | RKO |
| SK-PN-DW | MOG-G-UVW | HCC1937 | KYSE-140 | JHU-022 | SNU-175 |
| TC-71 | NMC-G1 | HCC1954 | KYSE-150 | KON | SNU-407 |
| U-2 OS | no-10 | HCC2157 | KYSE-180 | KOSC-2 cl3-43 | SNU-61 |
| **Brain** | no-11 | HCC2218 | KYSE-220 | LB771-HNC | SNU-C1 |
| 42-MG-BA | ONS-76 | HCC38 | KYSE-410 | OSC-19 | SNU-C2B |
| 8-MG-BA | PFSK-1 | HCC70 | KYSE-450 | OSC-20 | SW 1116 |
| A172 | SF126 | Hs 578T | KYSE-50 | PCI-15A | SW 1417 |
| AM-38 | SF268 | MCF7 | KYSE-510 | PCI-30 | SW 48 |
| Becker | SF-295 | MDA-MB-157 | KYSE-520 | PCI-38 | SW620 |

| SW837 | ARH-77 | U266B1 | NCI-H1092 | COR-L 105 | NCI-H1944 |
| --- | --- | --- | --- | --- | --- |
| T84 | ATN-1 | U-698-M | NCI-H1304 | EBC-1 | NCI-H1975 |
| WiDr | BALL-1 | **Liver** | NCI-H1417 | EKVX | NCI-H1993 |
| **Kidney** | BE-13 | C3A | NCI-H1436 | EMC-BAC-1 | NCI-H2009 |
| 769-P | C8166 | HLE | NCI-H146 | EPLC-272H | NCI-H2023 |
| 786-O | CMK | huH-1 | NCI-H1694 | H3255 | NCI-H2030 |
| A498 | CML-T1 | HuH-7 | NCI-H1836 | HARA | NCI-H2087 |
| A704 | DEL | JHH-1 | NCI-H187 | HCC-366 | NCI-H2228 |
| ACHN | EHEB | JHH-2 | NCI-H1876 | HCC-44 | NCI-H2291 |
| BB65-RCC | EJM | JHH-4 | NCI-H196 | HCC-78 | NCI-H23 |
| BFTC-909 | HC-1 | JHH-7 | NCI-H2081 | HCC-827 | NCI-H2342 |
| Caki-1 | HL-60 | SK-HEP-1 | NCI-H209 | HOP-62 | NCI-H2405 |
| CAL-54 | JVM-2 | SNU-182 | NCI-H2135 | IA-LM | NCI-H2444 |
| G-401 | JVM-3 | SNU-387 | NCI-H2141 | KNS-62 | NCI-H3122 |
| G-402 | K-562 | SNU-398 | NCI-H2171 | LC-1F | NCI-H322M |
| HA7-RCC | KARPAS-231 | SNU-423 | NCI-H2172 | LCLC-103H | NCI-H358 |
| KMRC-1 | KARPAS-620 | SNU-449 | NCI-H2196 | LK-2 | NCI-H441 |
| KMRC-20 | KMS-12-BM | SNU-475 | NCI-H345 | LOU-NH91 | NCI-H460 |
| LB1047-RCC | KU812 | **Lung** | NCI-H446 | LU65 | NCI-H520 |
| LB2241-RCC | L-363 | COLO-668 | NCI-H510A | LU99A | NCI-H522 |
| LB996-RCC | LC4-1 | COR-L279 | NCI-H524 | LXF-289 | NCI-H596 |
| NCC021 | LP-1 | COR-L303 | NCI-H526 | NCI-H1155 | NCI-H647 |
| OS-RC-2 | MEG-01 | COR-L311 | NCI-H64 | NCI-H1299 | NCI-H650 |
| RCC10RGB | MHH-CALL-2 | COR-L321 | NCI-H69 | NCI-H1355 | NCI-H661 |
| RCC-AB | MN-60 | COR-L88 | NCI-H841 | NCI-H1395 | NCI-H720 |
| RCC-ER | MOLP-8 | COR-L95 | NCI-H847 | NCI-H1437 | NCI-H727 |
| RCC-FG2 | NKM-1 | CPC-N | SBC-1 | NCI-H1563 | NCI-H810 |
| RCC-JF | NOMO-1 | DMS 114 | SBC-3 | NCI-H1568 | NCI-H838 |
| RCC-JW | OCI-AML2 | DMS 273 | SBC-5 | NCI-H1573 | PC-14 |
| RXF393 | OCI-AML5 | DMS 53 | SHP-77 | NCI-H1581 | RERF-LC-MS |
| SK-NEP-1 | P31/FUJ | H292 | SW 1271 | NCI-H1650 | SK-LU-1 |
| SN-12C | QIMR-WIL | HCC-33 | **Lung:NSCLC** | NCI-H1651 | SK-MES-1 |
| SW 13 | Reh | IST-SL1 | 201T | NCI-H1666 | SW 1573 |
| SW 156 | ROS-50 | IST-SL2 | A-427 | NCI-H1734 | SW 900 |
| TK10 | RPMI 8226 | LB647-SCLC | A549 | NCI-H1755 | UMC-11 |
| UO-31 | RPMI-8866 | LU-134-A | ABC-1 | NCI-H1781 |  |
| VMRC-RCW | SIG-M5 | Lu-135 | CAL-12T | NCI-H1792 |  |
| VMRC-RCZ | SK-MM-2 | LU-139 | Calu-3 | NCI-H1793 |  |
| **Leukemia** | SUP-T1 | LU-165 | Calu-6 | NCI-H1838 |  |
| ALL-PO | TALL-1 | MS-1-L | ChaGo-K-1 | NCI-H1915 |  |

| **Lymphoma** | RL | ACN | IOSE-523- | MIA PaCa-2 |
| --- | --- | --- | --- | --- |
| A3/KAW | RPMI 6666 | BE(2)-M17 | IOSE-75-16SV40 | Panc 02.03 |
| AMO-1 | SCC-3 | CHP-134 | JHOS-2 | Panc 04.03 |
| BC-1 | Sci-1 | GI-ME-N | JHOS-3 | Panc 10.05 |
| BL-41 | SLVL | GOTO | JHOS-4 | PANC-1 |
| CA46 | SR | IMR-32 | KGN | PA-TU-8902 |
| CTB-1 | SU-DHL-1 | IMR-5 | KURAMOCHI | PA-TU-8988T |
| Daudi | SU-DHL-10 | KELLY | OVCAR-3 | PL18 |
| DB | SU-DHL-16 | KP-N-YN | OAW28 | PL4 |
| DG-75 | SU-DHL-4 | KP-N-YS | OAW42 | PSN1 |
| EB2 | SU-DHL-5 | LAN-6 | OC-314 | QGP-1 |
| EB-3 | SU-DHL-6 | MHH-NB-11 | OV-17R | SU.86.86 |
| Farage | SU-DHL-8 | NB(TU)1-10 | OV-56 | SUIT-2 |
| GA-10 | SUP-HD1 | NB-1 | OV-7 | SW 1990 |
| GRANTA-519 | SUP-M2 | NB12 | OV-90 | YAPC |
| H9 | TK | NB13 | OVCA420 | **Pleura** |
| HDLM-2 | VAL | NB14 | OVCAR-4 | H2052 |
| HD-MY-Z | WIL2 NS | NB17 | OVCAR433 | H2369 |
| HH | WSU-DLCL2 | NB5 | OVCAR-5 | H2373 |
| Hs 445 | YT | NB6 | OVCAR-8 | H2461 |
| HT | **Miscellaneous** | NB7 | OVISE | H2591 |
| IM-9 | A388 | NBsusSR | OVK-18 | H2595 |
| Jiyoye | GCT | NH-12 | OVKATE | H2722 |
| JM1 | Hs 633T | SIMA | OVMIU | H2731 |
| JSC-1 | HT 1080 | SK-N-AS | OVTOKO | H28 |
| KARPAS-299 | JAR | SK-N-DZ | PA-1 | H2803 |
| KARPAS-422 | JEG-3 | SK-N-FI | PEO1 | H2804 |
| KM-H2 | MFH-ino | SK-N-SH | RKN | H2810 |
| L-1236 | SW684 | TGW | RMG-I | H2818 |
| L-428 | SW872 | **Ovary** | SK-OV-3 | H2869 |
| L-540 | SW982 | A2780 | SW 626 | H290 |
| MC/CAR | VA-ES-BJ | Caov-3 | TOV-112D | IST-MES1 |
| MC116 | **Muscle** | DOV13 | TYK-nu | MPP-89 |
| MHH-PREB-1 | A673 | EFO-21 | UWB1.289 | MSTO-211H |
| NU-DUL-1 | RD | EFO-27 | **Pancreas** | NCI-H2452 |
| OCI-LY-19 | RH-1 | ES-2 | CFPAC-1 | **Prostate** |
| P32/ISH | RH-18 | FU-OV-1 | Hs 766T | 22RV1 |
| Raji | RH-41 | Hey | HUP-T3 | DU 145 |
| Ramos-2G6-4C10 | SJCRH30 | IGROV-1 | KP-3 | VCaP |
| RC-K8 | **Nervous System** | IOSE-397 | KP-4 |  |

| **Skin** | SK-MEL-1 | **Thyroid** | **Uterus** |
| --- | --- | --- | --- |
| 451Lu | SK-MEL-2 | 8305C | AN3CA |
| A101D | SK-MEL-24 | 8505C | COLO 684 |
| A2058 | SK-MEL-28 | ASH-3 | EN |
| A-375 | SK-MEL-3 | B-CPAP | ESS-1 |
| A431 | SK-MEL-30 | BHT-101 | HEC-1 |
| A4-Fuk | SK-MEL-5 | CAL-62 | Ishikawa |
| C32 | UACC-257 | CGTH-W-1 | KLE |
| CHL-1 | UACC-62 | FTC-133 | MES-SA |
| COLO 792 | VMRC-MELG | HTC-C3 | MFE-280 |
| COLO-679 | WM-115 | IHH-4 | MFE-296 |
| COLO-783 | WM1552C | K5 | MFE-319 |
| COLO-800 | WM278 | ML-1 | RL95-2 |
| COLO-829 | WM793B | RO82-W-1 | SKN |
| CP50-MEL-B | **Stomach** | S-117 | SK-UT-1 |
| CP66-MEL | AGS | TT2609-C02 | SNG-M |
| G-361 | ECC10 | **UrinaryTrack** | **Vulva** |
| GAK | ECC12 | 5637 | CAL-39 |
| G-MEL | FU97 | 1A6 | SK-LMS-1 |
| HMVII | GCIY | 639-V | SW962 |
| Hs 939.T | GT3TKB | 647-V |  |
| Hs 940.T | HGC-27 | BFTC-905 |  |
| Hs 944.T | Hs 746T | CAL-29 |  |
| HT-144 | KATO III | HT 1376 |  |
| IGR-1 | MKN1 | J82 |  |
| IGR-37 | MKN28 | KU-19-19 |  |
| IPC-298 | MKN7 | LB831-BLC |  |
| IST-MEL1 | NCI-N87 | RT-112 |  |
| LB2518-MEL | NUGC-3 | RT4 |  |
| LOXIMVI | NUGC-4 | SCaBER |  |
| M-14 | RF-48 | SW 780 |  |
| MEL-HO | SCH | SW-1710 |  |
| MEL-JUSO | SNU-1 | T24 |  |
| MEWO | SNU-5 | TCCSUP |  |
| MMAC-SF | **Testes** | UM-UC-3 |  |
| MZ2-MEL. | NCC-IT-A3 | VM-CUB1 |  |
| MZ7-mel | NEC8 |  |  |
| RPMI-7951 | NTERA-S-cl-D1 |  |  |
| RVH-421 |  |  |  |
| SH-4 |  |  |  |
