# Supplementary material for: BRAF and AXL oncogenes drive RIPK3 expression loss in cancer
Source: PLoS Biol. 2018 Aug 29;16(8):e2005756. doi: 10.1371/journal.pbio.2005756 (PMC6114281; doi:10.1371/journal.pbio.2005756)
Supplement: S2 Table — (DOCX) [file pbio.2005756.s010.docx]

**S2 Table. Cancer cell lines sensitive to TSZ-induced necroptosis.**

| 23132/87 | ES4 | HSC-3 | MKN45 | OE21 | SW954 |
| --- | --- | --- | --- | --- | --- |
| AsPC-1 | GP5d | HT-29 | MUTZ-1 | Panc 03.27 | T.T |
| BC-3 | GR-ST | HUP-T4 | MZ1-PC | PC-3 [JPC-3] | TE-9 |
| BT-20 | H513 | JHH-6 | NB10 | RCM-1 | TOV-21G |
| BxPC-3 | HCC-15 | KY821 | NCI-H1688 | RERF-LC-KJ | TT |
| Capan-1 | HCC-56 | KYSE-270 | NCI-H2126 | SNU-1040 | TUR |
| CL-11 | HCE-4 | LCLC-97TM1 | NCI-H2170 | SNU-81 | U-937 |
| COR-L23 | HDQ-P1 | LS180 | NCI-H226 | SNU-C5 | WM35 |
| DAN-G | HPAC | LS-513 | NCI-H2347 | SW 1463 |  |
| EGI-1 | HPAF-II | MDA-MB-415 | OCUM-1 | SW-948 |  |
