# Supplementary material for: BRAF and AXL oncogenes drive RIPK3 expression loss in cancer
Source: PLoS Biol. 2018 Aug 29;16(8):e2005756. doi: 10.1371/journal.pbio.2005756 (PMC6114281; doi:10.1371/journal.pbio.2005756)
Supplement: S3 Table — (DOCX) [file pbio.2005756.s011.docx]

**S3 Table. Summary of statistical methods used to interrogate the expression levels of AXL, TYRO3 and RIPK3 in necroptosis resistant (NR) and necroptosis-sensitive (NS) cell lines, the correlation of these levels with necroptosis sensitivity (TSZ-IC50 values) and correlation with RIPK3 expression.**

| **NR vs. NS tests (N=941)** | **RIPK3** | **AXL** | **TYRO3** |
| --- | --- | --- | --- |
| Kolmogorov-Smirnov | <0.0001 | <0.0001 | 0.068 |
| Mann-Whitney | <0.0001 | 0.0002 | 0.023 |
|  |  |  |  |
|  |  |  |  |
| **Correlation vs. TSZ-IC50 (N=941)** | **RIPK3** | **AXL** | **TYRO3** |
| Pearson coefficient | -0.43 | 0.21 | 0.1 |
| Pearson p-value | <0.0001 | <0.0001 | 0.017 |
| Spearman coefficient | -0.23 | 0.18 | 0.021 |
| Spearman p-value | <0.0001 | <0.0001 | NS |
|  |  |  |  |
|  |  |  |  |
| **Correlation vs. RIPK3 expression (N=1018)** | **RIPK3** | **AXL** | **TYRO3** |
| Pearson coefficient | 1 | -0.26 | -0.11 |
| Pearson p-value | 0 | <0.0001 | 0.00058 |
| Spearman coefficient | 1 | -0.23 | -0.15 |
| Spearman p-value | 0 | <0.0001 | <0.0001 |
