# Supplementary material for: BRAF and AXL oncogenes drive RIPK3 expression loss in cancer
Source: PLoS Biol. 2018 Aug 29;16(8):e2005756. doi: 10.1371/journal.pbio.2005756 (PMC6114281; doi:10.1371/journal.pbio.2005756)
Supplement: S4 Table — (DOCX) [file pbio.2005756.s012.docx]

**S4 Table. Numbers of mutations found in necroptosis-resistant (NR, fully resistant) and necroptosis-sensitive (NS) cell lines for oncogenes significantly enriched in the necroptosis-resistant cell lines (i.e. Z-score >2).**

| **Oncogenes** | **Z-score** | **NR** | **NS** | **Enrichment** |
| --- | --- | --- | --- | --- |
| BRAF.MC | 4.78 | 24 | 0 | 24 |
| BRAF.V600E | 3.00 | 34 | 2 | 17 |
| YES1 | 4.78 | 24 | 1 | 24 |
| MET | 2.75 | 16 | 1 | 16 |
| RUNX2 | 2.49 | 15 | 0 | 15 |
| LCK | 2.49 | 15 | 1 | 15 |
| GLI1 | 2.24 | 14 | 1 | 14 |
