# Supplementary material for: BRAF and AXL oncogenes drive RIPK3 expression loss in cancer
Source: PLoS Biol. 2018 Aug 29;16(8):e2005756. doi: 10.1371/journal.pbio.2005756 (PMC6114281; doi:10.1371/journal.pbio.2005756)
Supplement: S5 Table — (DOCX) [file pbio.2005756.s013.docx]

**S5 Table. Numbers of mutations found in necroptosis-resistant (NR, fully resistant) and necroptosis-sensitive (NS) melanomas. Number of cell lines in each group and percentages of all analyzed melanoma cell lines are indicated. 69% of BRAF-mutant melanomas are fully resistant to TSZ-induced necroptosis.**

| 2 (3.6%) | 15 (27.2%) | 38 (69.1%) |
| --- | --- | --- |
| **NS** | **NR - BRAF WT** | **NR - BRAF MUT** |
| DJM1 (BRAF WT) | A431 | 451LU |
| WM35 (V600E) | A4FUK | A101D |
|  | CHL1 | A2058 |
|  | COLO792 | A375 |
|  | CP66MEL | C32 |
|  | GAK | COLO679 |
|  | GMEL | COLO783 |
|  | HS940T | COLO800 |
|  | HS944T | COLO829 |
|  | IPC298 | CP50MELB |
|  | LB2518MEL | G361 |
|  | MELJUSO | HMVII |
|  | MEWO | HS939T |
|  | MZ2MEL | HT144 |
|  | SKMEL2 | IGR1 |
|  |  | IGR37 |
|  |  | ISTMEL1 |
|  |  | LOXIMVI |
|  |  | M14 |
|  |  | MELHO |
|  |  | MMACSF |
|  |  | MZ7MEL |
|  |  | RPMI7951 |
|  |  | RVH421 |
|  |  | SH4 |
|  |  | SKMEL1 |
|  |  | SKMEL24 |
|  |  | SKMEL28 |
|  |  | SKMEL3 |
|  |  | SKMEL30 |
|  |  | SKMEL5 |
|  |  | UACC257 |
|  |  | UACC62 |
|  |  | VMRCMELG |
|  |  | WM115 |
|  |  | WM1552C |
|  |  | WM278 |
|  |  | WM793B |
