# Supplementary material for: BRAF and AXL oncogenes drive RIPK3 expression loss in cancer
Source: PLoS Biol. 2018 Aug 29;16(8):e2005756. doi: 10.1371/journal.pbio.2005756 (PMC6114281; doi:10.1371/journal.pbio.2005756)
Supplement: S6 Table — (DOCX) [file pbio.2005756.s014.docx]

**S6 Table. Mutational status of the cancer cell lines employed in the xenograft study described in Figure 1b-c and the changes in RIPK3 expression levels between *in vivo* serial passage 1 to passage 10. Most of the cell lines that experience loss of RIPK3 expression from p1 to p10 have mutations that lead to BRAF or AXL overactivation (green). Expression groups “high” and “low” were stratified at 4^th^ quartile cut-off. Data was obtained from the COSMIC and CCLE databases. Tyro3 and Mertk are the members of the TAM kinase family (Tyro3, Axl, Mertk).**

| **Mutations / Expression levels** | **Cell line** | **p1/p10 fold in RIPK3 expression** |
| --- | --- | --- |
| High Hras | OVCAR-3 | **3.19** |
| High Mertk | HOP-62 | **3.18** |
| High Tyro3 | HuH-7 | **2.77** |
| High Axl | SN12C | **2.60** |
| No expression/mutation data | RXF 393 | **2.36** |
| High Mertk | AsPC-1 | **2.08** |
| No expression/mutation data | HOP-92 | **2.01** |
| No expression/mutation data | CCRF-CEM | **1.67** |
| BRAF V600E | UACC-626 | **1.56** |
| BRAF V600E | LOX IMVI | **1.55** |
| BRAF V600E | HT-29 | **1.54** |
| High Axl, Tyro3 | PC-3 | **1.48** |
| KRAS G12V | OVCAR-5 | **1.45** |
| No expression/mutation data | SR | **1.43** |
| No BRAF mutation, no TAM high | MCF7 | **1.42** |
| No expression/mutation data | EKVX | **1.42** |
| High Axl | CAKI-1 | **1.41** |
| No expression/mutation data | HeLa | **1.40** |
| BRAF V600E | KM12 | **1.40** |
| No expression/mutation data | CA46 | **1.37** |
| No expression/mutation data | GTL-16 | **1.25** |
| KRAS G13D, High Tyro3 | HCT-15 | **1.25** |
| KRAS G13D, High Tyro3 | HCT-116 | **1.14** |
| KRAS A146T | HCC-2998 | **1.13** |
| No expression/mutation data | CP70 | 1.09 |
| BRAF V600E | COLO 205 | 1.09 |
| No expression/mutation data | AS283 | 1.08 |
| High Mertk, Yes, FGFR1 | NCI-H522 | 1.06 |
| KRAS G12S | A549 | 1.06 |
| No BRAF mutation, no TAM high | MDA-N | 1.05 |
| No BRAF mutation, no TAM high | HL-60 | 1.05 |
| Mertk high | MDA-MB-231 | 1.04 |
| No BRAF mutation, no TAM high | PC-3 | 1.01 |
| Mertk high | MDA-MB-435 | 1.00 |
| No BRAF mutation, no TAM high | NCI-H460 | 0.93 |
| No BRAF mutation, no TAM high | MOLT-4 | 0.92 |
| No BRAF mutation, no TAM high | U251 | 0.91 |
| No BRAF mutation, no TAM high | A549 | 0.86 |
| No BRAF mutation, no TAM high | K-562 | 0.85 |
| No BRAF mutation, no TAM high | SW-620 | 0.84 |
| BRAF V600E | MALME-3M | 0.83 |
| No BRAF mutation, no TAM high | 786-0 | 0.82 |
| BRAF V600E | COLO 829 | 0.76 |
| No BRAF mutation, no TAM high | M14 | 0.67 |
| BRAF V600E | A375 | 0.64 |
| No BRAF mutation, no TAM high | NCI-H226 | 0.59 |
| No BRAF mutation, no TAM high | NCI-H23 | 0.46 |
