# Supplementary material for: BRAF and AXL oncogenes drive RIPK3 expression loss in cancer
Source: PLoS Biol. 2018 Aug 29;16(8):e2005756. doi: 10.1371/journal.pbio.2005756 (PMC6114281; doi:10.1371/journal.pbio.2005756)
Supplement: S7 Table — (DOCX) [file pbio.2005756.s015.docx]

| **S7 Table. List of datasets used in this study.** | |
| --- | --- |
|  |  |
| **Cancer Type** | **GEO Profile / Database / Publication** |
| Gastric | GDS5336 / ILMN_2750356; Zhao CM et al. ***Sci Transl Med*** 2014 6(250):250ra115 |
| Colorectal | GDS4367 / 1448449_at; Tang A et al. ***Carcinogenesis*** 2012 Jul;33(7):1375-83 |
| Ovarian | GDS3894 / 1448449_at; Creekmore AL et al. ***PLoS One*** 2011 Mar 3;6(3):e17676 |
| Prostate | GDS2546 / 54622_at; Chandran UR et al. ***BMC Cancer*** 2007 Apr 12;7:64 |
| Breast | Ma XJ et al. ***Proc Natl Acad Sci U S A.*** 2003 100(10): 5974–5979 |
| Adrenocortical | cBioportal database |
| xenografts | GSE48433 Hollingshead MG et al. ***BMC Genomics*** 2014 May 22;15:393 |
